# Molecular insights into region-specific sexual dichromatism: Comparative transcriptome analysis of red cheek pigmentation in zebra finches

**DOI:** 10.1371/journal.pgen.1011693

**Published:** 2025-05-12

**Authors:** Gee-Way Lin, Chih-Kuan Chen, Ting-Xin Jiang, Ya-Chen Liang, Pin-Chi Tang, Ping Wu, Randall B. Widelitz, Chih-Feng Chen, Cheng-Ming Chuong

**Affiliations:** 1 Department of Pathology, Keck School of Medicine, University of Southern California, Los Angeles, California, United States of America; 2 Department of Biochemistry and Molecular Cell Biology, School of Medicine, College of Medicine, Taipei Medical University, Taipei, Taiwan; 3 The iEGG and Animal Biotechnology Center, National Chung Hsing University, Taichung, Taiwan; 4 Department of Animal Science, National Chung Hsing University, Taichung, Taiwan; University of Bern Faculty of Veterinary Medicine: Universitat Bern Vetsuisse Fakultat, SWITZERLAND

## Abstract

Feathers, the primary skin appendage covering the avian body, undergo dynamic phenotypic changes throughout a bird’s life. Males and females of the same species can exhibit sexually dichromatic plumage colors which play a critical role in mating choice, survival, and ecological interactions. In this study, we investigate the molecular mechanisms underlying the changes of color that occur during the transition from juvenile to adult feathers, known as the secondary transition. We focus on sexual dichromatism of craniofacial plumage and use the male cheek domain of the zebra finch (*Taeniopygia guttata*) as the major model. The transcriptome of the cheek and scalp (crown) domains in males and females of wild-type and genetic color variants were compared. We found that (1) Craniofacial color patterning operates through two regulatory layers. The first layer involves transcription factor (TF) genes that define the cheek domain such as PITX1, PAX1, PAX6. The second layer comprises pigment-related genes responsible for specific colors, including male-biased TFs (SOX10 and DMRT1) and transporters associated with red pigment synthesis. (2) Surprisingly, *ASIP*, which controls pheomelanin production in other species, was expressed in both male (red) and female (gray) cheeks. Instead, *PAX1* in cheek dermal fibroblasts may serve as an upstream regulator, potentially triggering the male-biased color pattern through PAX6 and SOX10. PAX6 and SOX10 in melanocytes potentially enhance the expression of *GPR143*, *SLC45A2*, and *TMEM163*, driving increased pheomelanin production in males. (3) Sexual dichromatism is associated with sex-linked genes on the Z chromosome, notably *SLC45A2*. In addition, motif analysis comparing the binding strength between regional transcription factors and melanogenesis genes suggests that craniofacial pigmentation may have evolved convergently in passerine birds. These findings provide novel insights into the molecular control of color patterning and lay the groundwork for further studies on avian sexual dichromatism and secondary feather transition.

## Author summary

Passerine birds display complex, region-specific color patterns, often differing by sex and species. We wonder how these complex color patterns are established. We use the zebra finch as a model because of its region-specific sexual dichromatism. Zebra finch cheeks are red in males and gray in females and some genetic variants have different cheek colors. We then compare the cheek genes to those found in the scalp (or crown) that do not show sexual dichromatism. Through transcriptomic analyses, we identified transcription factors (TF genes) enriched in the cheek versus scalp domains, providing insights into the molecular drivers of localized pigmentation. Notably, we found several Z chromosome genes associated with the male red cheek color, providing evidence of sexual dimorphism under cell-autonomous genetic control. Agouti-signaling protein (ASIP) expression in both male red and female gray cheek feathers imply ASIP has a broader role in feather development beyond pheomelanin production. While craniofacial domains are conserved in the passerine (primary transition), comparative TF motif analysis across passerine species reveals that color diversification has evolved convergently (secondary transition).

## Introduction

**Fig 1:**
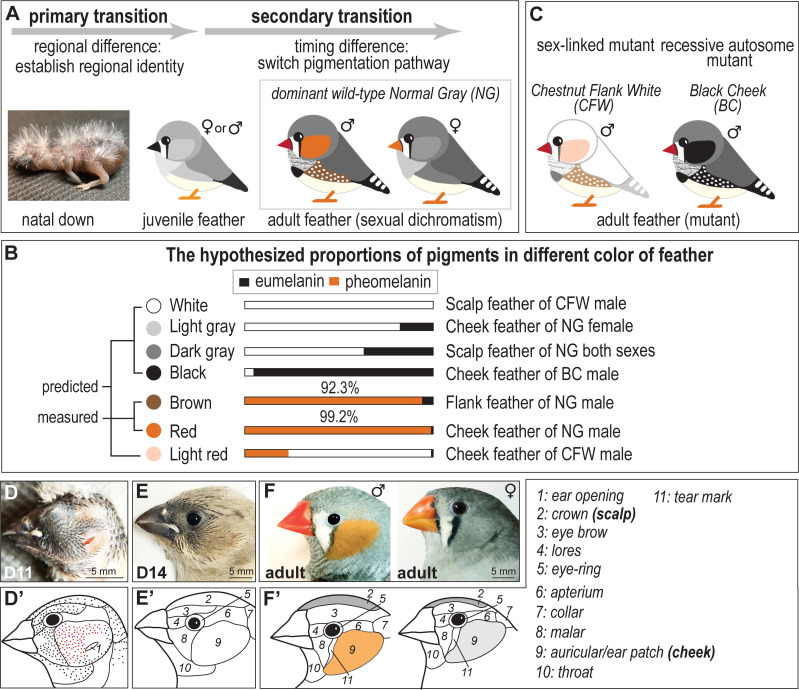
Temporal development of sex dichromatic feathers in craniofacial domains of zebra finch. (A) Scheme of the two stages of feather transitions from natal downs to adult feathers. The primary feather transition pertains solely to immature plumage exhibiting a gradient of gray [5]. Regional domains are established during embryonic development and maintained through juvenile stages. The secondary feather transition leads to the emergence of sex dichromatic plumage through a shift in the pigmentation pathway during the replacement of juvenile male or female feathers with mature feathers. (B) The predicted and hypothetical concentrations of pheomelanin and eumelanin in seven colors of zebra finch plumage. The red cheek feathers and brown flanking feathers of males are predicted to be primarily composed of pheomelanin pigments (over 90%) [31], although eumelanin pigments are also present. This suggests that the production of pheomelanin and eumelanin is not entirely exclusive within a single feather. In other words, a higher proportion of pheomelanin results in redder feathers, while a higher proportion of eumelanin results in darker, blacker feathers. (C) Two types of plumage variants arise from mutations. Chestnut Flank White (CFW), characterized by lighter red cheek coloring, is predicted by breeders to result from mutations on the sex chromosomes. Black Cheek (BC), characterized by black cheek coloring, is predicted to result from autosomal mutations. (D–F) Secondary feather transition, marking the transition from juvenile to adult feather forms. (D) 11-day-post-hatching (D11) juveniles with clear feather tracts and naked skin. (E) Craniofacial skin of 14-day-post-hatching (D14) juveniles is completely covered by feathers. (F) Craniofacial plumage of mature wild-type adult finches. Male with bright red cheek patch is shown in the left panel, while female with cryptic gray cheek patch is shown in the right panel. (D’–F’) Feather tract domain boundaries seen in (D–F) are delineated, and the specific name of each domain is listed. The crown domain is referred to as scalp and the ear patch domain termed as cheek in this study.

Patterns are essential to many fundamental biological functions [1]. Among them, plumage color patterns play a critical role in avian social interactions including mate choice [2]. Feather color patterns change throughout a bird’s life, enabled by the evolution of stem cell-based follicles, which allow for cyclic molting and feather renewal [3]. Feather molting refers to the periodic process of shedding and regenerating feathers in a single follicle. Beyond the feather molting, broader phenotypic changes in plumage occur throughout a bird’s life—a process known as feather transition. This process provides adaptive advantages, enabling birds to reset feather phenotypes in response to changing environments at different life stages [4]. In a bird’s life, two stages of feather transitions are typically observed. The primary transition, from natal down to juvenile feathers (Fig 1A, 1D and 1E), establishes the basic contour feather structures and is regulated by conserved molecular mechanisms across bird species, ranging from the chicken (*Gallus gallus*), a precocial Galliform bird, to the zebra finch (*Taeniopygia guttata*), an altricial Passeriform bird [5]. However, adult birds exhibit remarkable diversity in feather forms, colors, and rigidity, largely shaped by phenotypic changes that occur after juvenile, a process referred to as the secondary feather transition (Fig 1A) [5].

Male and female avian species often experience different selection pressures that give rise to sexually dimorphic traits (size, mass, shape, colors, song, and behavior) [6,7]. Plumage sexual dichromatism emerge during the secondary feather transition and is driven by hormonal regulation, cell-autonomous genetic influence (e.g., sex chromosomes), or a combination of both [8–12]. In a previous study using black-feathered Taiwan country chickens, we showed that hormones might contribute to regional specificity and pigmentation patterns. First, sex-dimorphic saddle feathers were perturbed by hormone treatments [13]. Second, male saddle feathers were switched to female color patterns after estradiol injection [13]. However, in zebra finches, early castration of males does not prevent the development of normal male plumages [14], even though juvenile testosterone levels are significantly lower and plumage maturation is delayed [15,16]. Another study focusing on zebra finch gynandromorphs shows that their plumage color pattern aligns more with the genetic sex (sex chromosome) despite of the gonad sex [17]. These results suggest that plumage sexual dimorphism can be affected by sex chromosome-dependent cell-autonomous mechanism or by gonad-dependent sex hormone mechanism.

While the mechanisms underlying spatial color domains across the body remain largely unknown, previous studies have explored color patterning from organismal and evolutionary perspectives [18,19]. More recently, efforts have linked color patterns to developmental processes. For example, the longitudinal color stripes of juvenile galliform birds are based on somite pre-patterning [20], and melanocytes provide instructive patterning signals [21]. In finches, a comprehensive study of complex plumage patterns on the trunk revealed conserved body color domains linked to embryonic skin regions, demonstrating that the embryonic prepattern is largely conserved among birds despite significant adult color variation [22].

Plumage diversity and the recognition of color signals are critical factors in speciation [23]. Conspicuous craniofacial plumage colors used in visual communication have been observed in species like *Passerina* manakins and various rainforest birds [24,25]. A broader analysis of 500 Australian bird species showed that sexual dichromatism is primarily concentrated in the head domain [26]. Thus, the domesticated passerine zebra finch (*T. guttata*) is an excellent model for studying these molecular mechanisms due to its distinct sexually dimorphic cheek patches. Zebra finch behavioral studies showed that the brightness of male beak color significantly influences female mate choice [27–29]. However, the cellular and developmental mechanisms that control craniofacial plumage color patterning remain unexplored.

In this study, we focus on identifying the molecular factors involved in the sexually dichromatic cheek domain plumage of the zebra finch (*T. guttata*), which emerge through the secondary feather transition. Feather color is determined by pigments and the nanostructure within feather barbs or barbules [30]. Sexual dichromatism in many finches has been suggested to arise through a common mechanism involving the degradation of integumentary carotenoid pigments [9]. However, the zebra finch plumage colors are melanin-based rather than carotenoid-based pigments, which constitute less than 0.005% of carotenoid-based pigments [31,32]. The chemical pigments in the male red cheek and brown flanking feathers are primarily attributed to pheomelanin (Fig 1B), which is synthesized by melanocytes [31]. To characterize the phenotypes underlying sex-biased zebra finch melanogenesis, we collected the cheeks of dominant wild-type males and females (with red and gray cheeks, respectively), a recessive autosomal mutant (with black cheeks in both sexes), and a sex-linked mutant (with light red cheeks in males) (Fig 1A and 1C). We propose that hypothesized proportions of pheomelanin and eumelanin pigments in seven plumage colors (white, light gray, dark gray, black, brown, red, and light red) observed in wild-type and mutant zebra finches (Fig 1B). To uncover the underlying molecular mechanisms controlling sex biased pigmentation, we examined developmental spatial domains of dichromatic cheeks versus non-dichromatic scalps from juveniles to adults, conducted transcriptome analyses of these domains in wild types and mutants, and investigated sex chromosome-linked gene expression. Finally, we examined the links between the transcriptional signals and pigmentation genes and conducted a cross-species motif analysis to decipher evolutionary patterns in passerines. These findings uncover unexpected results and offer new insights into the study of sexual dichromatism, craniofacial regional specification, color regulation and secondary feather transition in a bird’s life.

## Results

### Development of sexual dichromatism in craniofacial plumage of zebra finches across life stages

Birds are the most diverse group of terrestrial vertebrates, a distinction largely attributed to the remarkable variation in feather forms, colors, and rigidity. Upon reaching sexual maturity, male zebra finches (*T. guttata*) undergo physiological changes, typically driven by hormonal effects, which transform skin appendages such as the feathers covering the ear holes (cheek patches) and skin covering beak into red color. This feather change is referred to as the secondary transition (Fig 1A, 1E and 1F, left panel). In contrast, female zebra finches retain cryptic color patterns of white, gray, and black, which are similar to their juvenile plumage (Fig 1E and 1F, right panel).

Focusing on the zebra finch head domains, feather tract growth was documented through lateral views of juveniles from 1-day to 14-days old (D1 to D14 in Figs S1A, 1D and 1E). Most feather tracts in the lateral view of juveniles become distinctly visible by 11 days (Fig 1D). Each individual feather follicle at D11 was represented as a dot, with the auricular tracts specifically highlighted in red (Fig 1D’). To illustrate the potential boundaries of these feather tracts, each domain was delineated with a line (Fig 1D’and 1E’). The cheek domain size in zebra finches is considerably larger than in basal avian species such as chickens and pigeons (Figs 1F’, S2A and S2B). Additionally, the zebra finch cheek domain expands anteriorly, resulting in a smaller malar domain compared to that of chickens and pigeons (Figs 1F’, S2A and S2B). The feathers covering the finch cheek domain are mainly composed of opercular feathers originating from the anterior auricular tracts (red spots in Fig 1D’), rather than auricular feathers from the posterior auricular tracts (S2A’ Fig). The opercular feathers collected from the cheek domain (also called cheek feathers in this study) are smaller in size and possess a sharper shape compared to other craniofacial feathers, such as the scalp (crown) feathers located at the top of the head (S1B and S1C Fig). During the secondary feather transition, craniofacial skin domains are established, equivalent to the primary feather transition process [5].

Based on our morphological observation, establishing sexual dichromatism in the zebra finch craniofacial plumage may involve two processes. 1) Pattern establishment: During the primary feather transition, the auricular tracts cannot be identified on the skin until at 4 days post-hatching (D4), with the anterior auricular tracts first observed at 7 days post-hatching (D7) (S1A Fig). The ear holes serve as initial signaling centers for auricular tracts, giving rise to the finch cheek domain. This regional cheek domain identity is established during embryogenesis and maintained in juveniles as their immature plumage develops during the primary feather transition (Fig 1A) [5]. 2) Sexual dimorphism is under cell-autonomous genetic regulation: In wild-type zebra finches, sexually dimorphic plumage color patterns emerge during the secondary feather transition (Fig 1A and 1F). The sex-linked mutation, Chestnut Flank White, shown in Figs 1C and S1D, serves as an example suggesting that male cheek color may be governed by sex-linked genes. Additionally, the shift in the pigmentation synthesis pathway represents a downstream target of the regulatory hierarchy of sex-linked genes.

### Analysis of cheek and scalp (crown) transcriptomes in zebra finch variants

**Fig 2:**
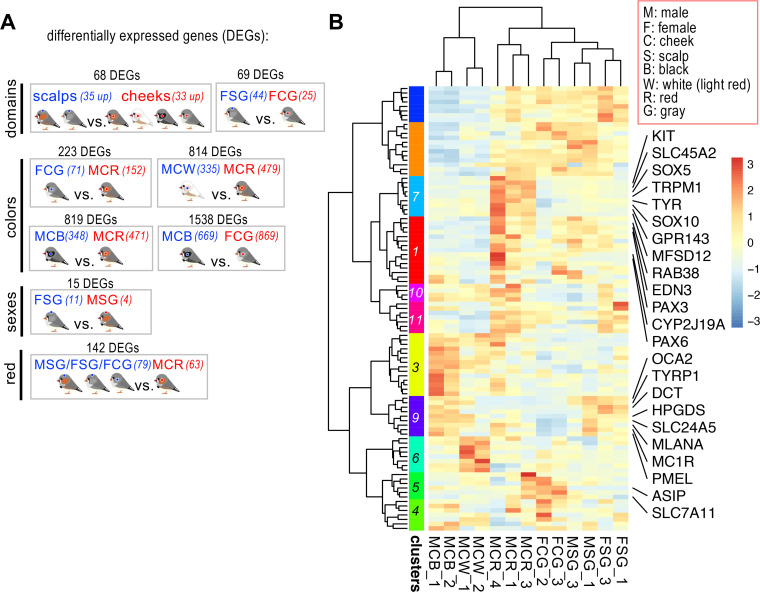
Transcriptome analyses reveal differentially expressed melanogenesis genes. (A) The comparisons between different domains (scalp and cheek), cheek feather colors, sexes, and between male cheek red (MCR) feathers and others are exhibited, respectively. The number of differentially expressed genes (DEGs), exhibiting statistically significant changes (padj < 0.05, log2 Fold Change > 1 or < −1), in each panel is indicated. The significant DEGs for each comparison are listed in S1 File. (B) Hierarchical clustering and heatmap representation of DEGs derived from a melanogenesis gene list (S2 File). DEGs in each cluster are listed in S3 File. Abbreviations of sample name and axes are highlighted in red boxes.

To identify the genes responsible for changes in cheek color, we searched for differentially expressed genes (DEGs) in the cheeks of male and female wildtype zebra finches and their species variants, such as Chestnut Flank White (CFW) with light red cheeks and Black cheek (BC) displaying black cheeks. As a control, we also performed transcriptome analysis on scalp feathers which use muted colors with less variation between individuals (Fig 2A). In total, eight comparison groups were examined, encompassing domain, color and sex differences (Fig 2A). Volcano plots and DEGs from each comparison are presented in S3 Fig and S1 File. The integration of these comparisons generated a total 2,462 unique color-associated DEGs. The number of DEGs for male cheek red (MCR) versus male cheek white (MCW) and male cheek black (MCB) are 814 and 819, respectively (Fig 2A). Interestingly, both are significantly higher than the number of DEGs for MCR versus female cheek gray (FCG), which is 223 (Fig 2A). We conducted gene set enrichment analysis on each DEG set and identified that extracellular matrix structural constituent and cell adhesion molecule binding were strongly enriched in the first two comparisons, suggesting that the formation of light red (MCW) and black (MCB) cheeks is not only restricted to pigmentation pathways but also involve genes related to tissue microstructures (S1 File).

To evaluate the roles of known color genes in finch cheeks, we first intersected the color-associated DEGs with a compiled list of genes associated with the melanogenesis pathway (496 genes) and the carotenoid pathway (17 genes), as reported from previous studies (S2 File). We then conducted hierarchical clustering to group the color genes based on their expression profiles across samples, focusing on genes upregulated in red cheeks (clusters 1 and 7, Fig 2B and S3 File). Most of these genes belong to the melanogenesis pathway rather than the carotenoid pathway, consistent with previous findings [32]. Notably, while pheomelanin synthesis genes were up-regulated in male cheek feathers (clusters 1 and 7), eumelanin synthesis-related genes (cluster 9) were down-regulated in female cheek feathers (Fig 2B). Interestingly, *ASIP* is enriched in all cheek feather colors—ranging from red, light red, gray, and even black—in contrast to scalp feathers (cluster 5, Fig 2B).

### Identifying potential transcriptional regulators for the cheek domain

**Fig 3:**
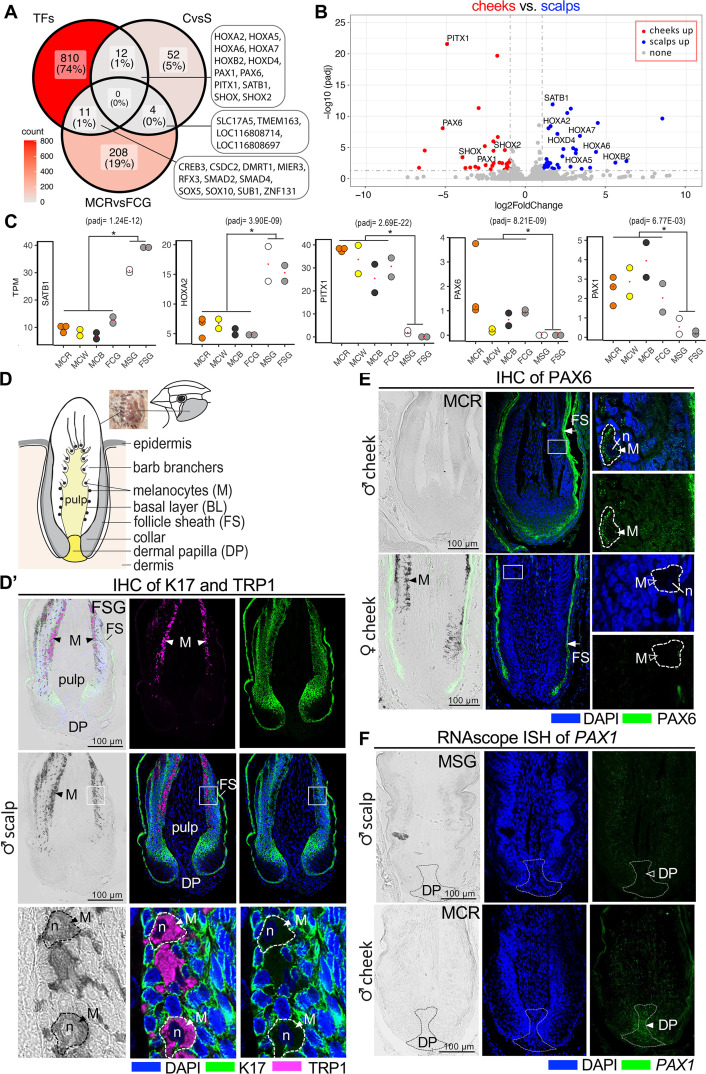
Region-specific transcription factor (TF) genes of cheek and scalp feathers. (A) To rule out the sex effect of transcription factors in our dataset, we show the number of candidates for each individual dataset (CvsS, MCRvsFCG and TFs) and highlight the candidate DEGs found at the intersection of all three datasets. (B) A volcano plot depicting the 12 differentially expressed regional TF genes upregulated in the cheek (red) and scalp domain (blue). (C) Representative genes *SATB1* and *HOXA2* exhibit high TPM levels in scalps, whereas representative genes *PAX1*, *PAX6* and *PITX1* display high TPM levels in cheeks. Asterisks mark the statistically significant difference between groups (padj < 0.05, log2 Fold Change > 1 or <−1). (D) Illustration of cheek patches in zebra finch and the longitudinal section of a single growing feather. For this study, the growing feather of the anterior ear cover, also known as the operculate feather from the auricular tract [34] were used for analysis. For the longitudinal section, the dermis (tan) and epidermis layers (gray and white) are shown. The feather follicle is wrapped with various epidermis layers including the follicle sheath (FS), intermediate layer, and basal layer (BL). The pulp contains a central axial artery and numerous proliferating mesenchymal cells. The melanocytes are distributed along the basal layer (black). At the base of the feather follicle lies the dermal papilla (yellow). (D’). Immunohistochemistry using K17 and TRP1 antibodies. The left upper panel shows an image merged from four channels, including bright field to display the eumelanin pigments (black), DAPI staining to show the nuclei (blue), keratin staining to reveal cell morphology (green), and TRP1 staining to indicate the enzyme involved in melanin synthesis within melanocytes (M) near the basal layer (magenta). In the bottom panels, the larger and more irregular size of melanocytes near the basal layer is magnified and highlighted with dashed lines. The nuclei (n) of the melanocytes are also indicated. (E) Immunohistochemistry using PAX6 antibody. The left panels show the bright field, while the middle panels display merged images of nuclear staining with DAPI (blue) and the PAX6 antibody (green). The right panels provide magnified views from the middle channels, focusing on the intermediate layer containing melanocytes. PAX6 proteins are strongly expressed in the feather sheath (FS) of all feather follicles (arrows). However, only melanocytes (M) within male cheek feathers display PAX6 protein expression (arrowheads), in contrast to melanocytes within female cheek feathers (hollow arrowheads). (F) RNAscope analysis utilizing *PAX1* probes. Expression of *PAX1* mRNA is primarily enriched in the dermal region (DP, dermal papilla, dashed lines) of male cheek feathers.

To identify the regulators responsible for the cheek and scalp domains, we first compared male and female wildtype scalps with male and female wildtype cheeks and obtained 68 DEGs (Figs 2A and 3A, group CvsS and S1 File). We next compared our dataset with the number of reported transcription factors (TFs) in a published database (833; https://ngdc.cncb.ac.cn/databasecommons/database/id/8), and identified 12 TF genes that overlap between these two datasets (Fig 3A). To minimize the influence of color and sex, we examined the overlap between the 12 TFs and the DEGs from the MCR versus FCG comparison (223) and observed no overlaps (Fig 3A). A refined volcano plot was generated to contrast the cheek versus scalp domains, highlighting five TF genes in the cheek and seven TF genes in the scalp (Fig 3B). In the scalp domain, a chromatin organizer *SATB1* and several *HOX* genes exhibit greater enrichment compared to the cheek domain (Figs 3C and S4A). The major up-regulated TF genes in the cheek domain are *PITX1*, *PAX6* and *PAX1* (Fig 3B and 3C and S1 File), as confirmed through quantitative PCR (S4B Fig). Though *SHOX* and *SHOX2* showed higher expression levels in cheeks than in scalps, they were deprioritized for further analysis due to their low overall expression levels (Transcripts Per Kilobase Million [TPM] value < 1; S4 File).

We next examined the distribution of candidate transcription factors within cheek feathers using immunohistochemistry or RNAscope ISH. A schematic diagram of a longitudinal section is presented as a guide showing the positions of different cell types within the feather follicle (Fig 3D). We characterized cell morphology and the location of melanocytes within longitudinal sections using immunohistochemistry for several markers: keratins (K17 antibody), the enzyme for melanin synthesis (TRP1 antibody, also called TYRP1), and the marker for melanocytes (MITF antibody) (Figs 3D’, S5A and S5B). Notably, the melanocytes are the largest and most irregular cells, located near the basal layer of the epidermis (Figs 3D’ and S5B).

Among the TFs for scalp domain, SATB1 is the only one antibody produced from human antigens, and able to cross-react with zebra finch proteins (S4C Fig). In contrast, there are no available HOX antibodies produced from mouse or human antigens capable of specifically detecting HOX proteins in zebra finches. SATB1 proteins have been shown to play a role in higher-order chromatin remodeling [33]. They are prominently expressed in the nuclei of keratinocytes in longitudinal sections of scalp feathers compared to cheek feathers in both males and females (S4C Fig), suggesting they may play a role in regulating downstream gene expression.

In the cheek domain, pheomelanin pigments (red) in the male cheek are more difficult to observe than eumelanin pigments (black) in the female cheek (Figs 3E, S5A and S5B). Since we found elevated *PAX6* expression levels in male cheeks (Fig 3B and 3C), we immunostained with anit-PAX6 antibodies and found it was located in cells with a larger and more irregular size located near the basal epidermal layer which is consistent with their being melanocytes (Figs 3D’ and S5B). Despite PAX6 protein being highly enriched in the feather sheaths of both sexes, PAX6 proteins are distributed within male cheek feather melanocytes (marked by dashed lines in the magnified panels) (Fig 3E). Similarly, *PAX1* mRNA shows male-biased enrichment in the dermal papilla of cheek feathers (Fig 3F). On the other hand, *PITX1* mRNA was randomly distributed in the cheek feather sections of both sexes, as shown by RNAscope ISH, but no signal was observed in the scalp feather sections (S4D Fig). We reasoned that PITX1 might be involved in initiating domain determination. Whole-mount *in situ* hybridization was used to detect the expression of *PITX1* mRNA in early finch embryos. While no specific signals for *PITX1* mRNA were observed at embryonic day 7 (E7), *PITX1* was detected in the developing feather buds surrounding the ear hole and eyebrows at E8 (S4E Fig). Collectively, we identified three TF genes, *PAX1*, *PAX6*, and *PITX1*, enriched in the cheek domain of both sexes. However, within the feather sections, *PAX1* transcripts were detected exclusively in the dermal papilla and PAX6 proteins exclusively in the melanocytes of male red feathers.

### Re-evaluating the roles of ASIP and pigment-producing melanocytes in pheomelanin pigmentation

**Fig 4:**
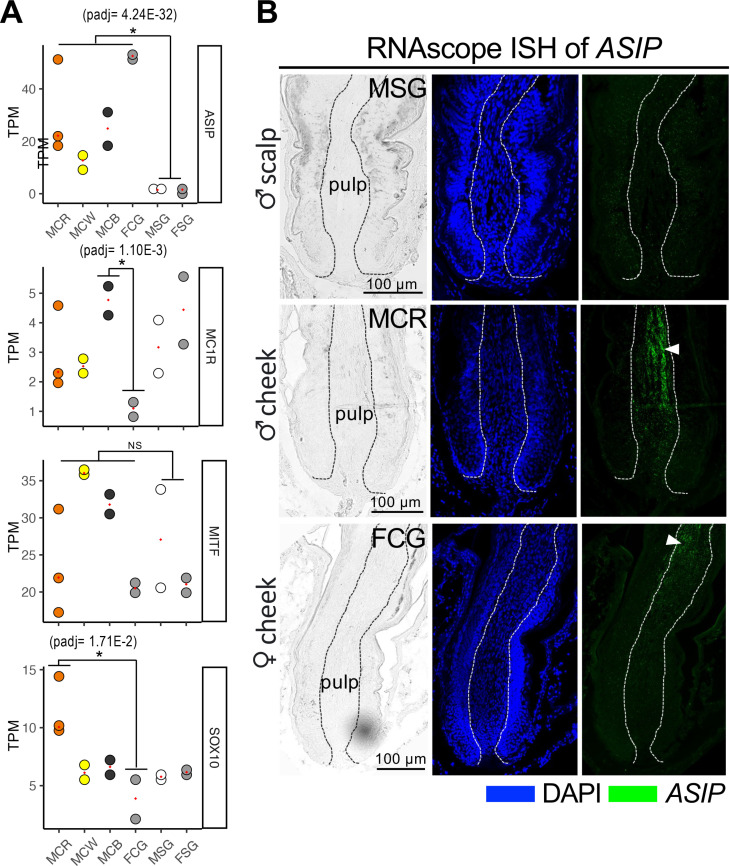
Expression of *ASIP* in cheek feather follicles of both male and female finches. (A) TPM values of representative genes involved in pigment production. *ASIP* exhibits high TPM levels in all cheeks. The TPM value of *MC1R* is highest in MCB and lowest in FCG, resulting in a significant difference between FCG and MCB. No significant differential TPM levels (NS) are observed for *MITF* across domains and colors. *SOX10* displays high TPM levels in male red cheek feathers. An asterisk marks the statistically significant difference between groups (padj < 0.05, log2 Fold Change > 1 or < −1). (B) RNAscope analysis utilizing *ASIP* probes. *ASIP* mRNA is enriched in the pulp region (indicated by arrowheads) in both male and female cheek feathers.

Conventionally, the interaction of ASIP (Agouti-signaling protein) with MC1R (Melanocortin 1 receptor) is known to reduce MC1R activity, thereby influencing melanocytes to produce pheomelanin instead of eumelanin. However, we unexpectedly discovered that *ASIP* is enriched in all colors of finch cheek feathers, regardless of the black cheeks (Fig 4A). All the validation results performed by qPCR and RNAscope ISH align with the transcriptome data, showing that *ASIP* mRNA is enriched in both red and gray wildtype cheek feathers compared to the scalp feathers (Figs 4B and S5C). In the longitudinal sections of cheek feathers, *ASIP* mRNA was identified in the central pulp regions of both red (male) and gray cheek feathers (female) (Fig 4B). Although *ASIP* mRNA seems to be distributed only in the upper part of the female cheek feather pulp, this may be due to different growth phases of the compared samples. Notably, no *ASIP* mRNA localization differences were detected between male and female scalp feathers (upper panel of Fig 4B). In previous studies, the association between MC1R polymorphisms and plumage coloring is not supported in passerine birds like zebra finches, flycatchers and leaf warblers [35]. In addition, SOX10 is recognized for its significant involvement in the establishment and proper functioning of melanocytes, as well as in eumelanin and pheomelanin synthesis [36,37]. Thus, we confirmed the expression levels of *MC1R*, *MITF* and *SOX10* through qPCR, revealing no significant differences of *MC1R* and *MITF* across all wild-type feathers (S5C Fig). We also analyzed the numbers, distributions, and differentiation status of melanocytes within feather follicles using MITF and SOX10 (melanocyte markers) and TRP1 antibodies (S5A, S5B and S5C Fig). In the center of longitudinal sections, no significant differences were observed in melanocyte numbers or distributions between male and female cheek feathers (S5A and S5B Fig). Although SOX10 was identified as being specifically elevated in male red cheek feathers based on RNAseq and qPCR validation (S5C Fig), SOX10 proteins were detectable in the nuclei of melanocytes in both red and gray cheek feathers (S5D Fig). In short, the detection of melanocytes, SOX10 proteins, and *ASIP* transcripts within the cheek feathers did not reveal any distinct patterns between males and females that could explain the color variation.

### Several genes located on sex chromosome Z are associated with the red colored male zebra finch cheek

**Fig 5:**
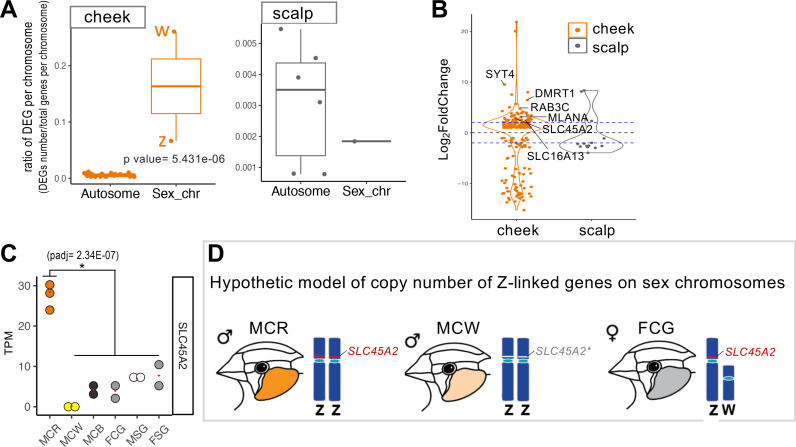
Sex-linked genes and sexual dichromatism in cheek feathers. (A) Ratios of sex chromosome DEGs are higher than those of autosomal DEGs in the cheek (orange box, comparing MCR vs. FCG) but not in the scalp (gray box, comparing MSG vs. FSG). Each dot represents the ratio for a chromosome. (B) DEGs on the Z chromosome with over four-fold higher expression in males are marked. A log2 fold change value above 0 indicates genes with higher expression in males, while a value below 0 indicates the higher expression in females. The blue lines indicate the value above 2 or below −2. The orange color indicates DEGs from the cheek comparison, while gray color indicates DEGs from the scalp comparison. (C) TPM levels of the sex-linked gene *SLC45A2* among the cheek colors of three variants (R: red, W: white, and B: black). An asterisk marks the statistically significant difference between groups (padj < 0.05, log2 Fold Change > 1 or < −1). (D) Sex chromosomes for male and female and a hypothetical model of sex-linked gene *SLC45A2* on Z chromosomes within the three variants of cheek colors. The saturation of red cheek color in MCR is closely related to high expression of *SLC45A2* from two copies. In contrast, the significantly reduced expression of *SLC45A2* in the light red cheek (MCW) from two copies, which is lower than that from a single copy in female gray cheek (FCG), may be resulted from mutation or negative regulation on *SLC45A2*.

The plumage phenotype in gynandromorph zebra finches consistently correlates with the genetic sex of the gonads, suggesting that sexual differentiation of zebra finch plumage is primarily governed by cell-autonomous genetic control [17]. To further test this hypothesis, we examined the chromosomal distribution of the 223 cheek DEGs associated with color difference (the MCR versus FCG comparison, Fig 2A) and the 15 scalp DEGs associated with sex difference (the MSG versus FSG comparison, Figs 2A, 5A and S6A). A high proportion of DEGs from the cheek comparison was found on the sex chromosomes (W and Z), but this difference was not observed in the scalp comparison (Figs 5A and S6A). We further focused on 71 DEGs highly expressed in the male cheek that are located on the Z chromosome. Of these, 53 DEGs result from the dosage effect (absolute log2 fold change around 1 to 1.5), while the remaining 18 DEGs exceed the dosage effect. *SYT4*, *DMRT1*, *RAB3C*, *MLANA*, *SLC45A2*, and *SLC16A13* from the 18 DEGs exhibit over four-fold higher expression (absolute log2 fold change > 2) in male cheek feathers compared female cheek feathers (Fig 5B).

The functions of *SYT4* (*Synaptotagmin 4*) in melanocytes have been revealed in recent years [38]. In alpaca melanocytes and melanoma cells, SYT4 overexpression regulates calcium influx through TRPM1 and promotes dendrite elongation [38,39]. TRPM1 forms ion channels on melanocytes and its cellular function in melanocytes is closely associated with melanin production [40]. In zebra finches, *TRPM1* is significantly enriched in male red cheeks (Figs 2B and S6B), suggesting its role in promoting melanin synthesis for red cheek feathers. *MLANA* (*Melan-A* or *MART1*) plays a critical role in melanosome biogenesis, particularly during the early stages [41]. It is crucial for controlling the expression, stability, trafficking, and processing of the melanocyte protein PMEL [42]. Additionally, MLANA cooperates with GPR143 to ensure proper melanosome composition [43]. Although *MLANA* is identified as one of the Z-linked DEGs exceeding the dosage effect threshold (Fig 5B), its expression level is specifically reduced in female gray cheeks but not in female scalps (Figs 2B and S6C and S4 File).

Another Z-linked DEGs *SLC45A2* is known to be expressed in melanocytes and functions as a transporter to regulate melanosome pH [44]. A genetic study in capuchinos seedeaters, a finch species with rapid male plumage radiation, revealed diversification in the regulatory regions of *SLC45A2*, suggesting that its regulation correlates with sexual dichromatism [45]. Moreover, the typical light-red cheek mutant, Chestnut Flank White (CFW) (Figs 1C and S1D), has been identified to be a Z-linked recessive mutant by the breeders. Our analysis shows that the expression level of *SLC45A2* in the light-red cheek mutant is reduced to a level similar to that in the gray cheek of wild-type female zebra finches (Fig 5C), suggesting the possibility that the Z-linked recessive mutant is related to the attenuation of *SLC45A2* for the lighter coloring (Fig 5D). Moreover, some missense *SLC45A2* mutations at the *Silver* locus in chickens are dominant and lead to pheomelanin dilution [46]. To summarize, sex-linked genes constituted a significant proportion (31.8%) of cheek DEGs between sexes. Among these genes, *SLC45A2* emerged as the most notable Z-linked gene with potential to promote pheomelanin deposition.

The DEGs on the W chromosome were also examined for their functions as they could play a role as negative regulators for red cheek coloring. Among the 43 DEGs on the W chromosome, 15 are uncharacterized genes (S1 File) Based on our literature review, no direct link was identified between the 28 annotated DEGs and hormone or melanogenesis regulation (S1 File). However, *ATP5F1A* encodes a subunit of the mitochondrial ATP synthase complex, which is involved in the production of adenosine 5’-triphosphate (ATP). ATP can stimulate melanogenesis, or melanin production, in melanocytes [47]. In addition, *ZFAND5* was predicted to act upstream of or within several processes, including face development [48]. Therefore, although the DEGs on the Z chromosome are highly related to red cheek coloring, we do not rule out the possibility that W-linked genes can also regulate melanogenesis.

### Exploring potential links between the cheek-regional transcription factors and the pigmentation pathways

**Fig 6:**
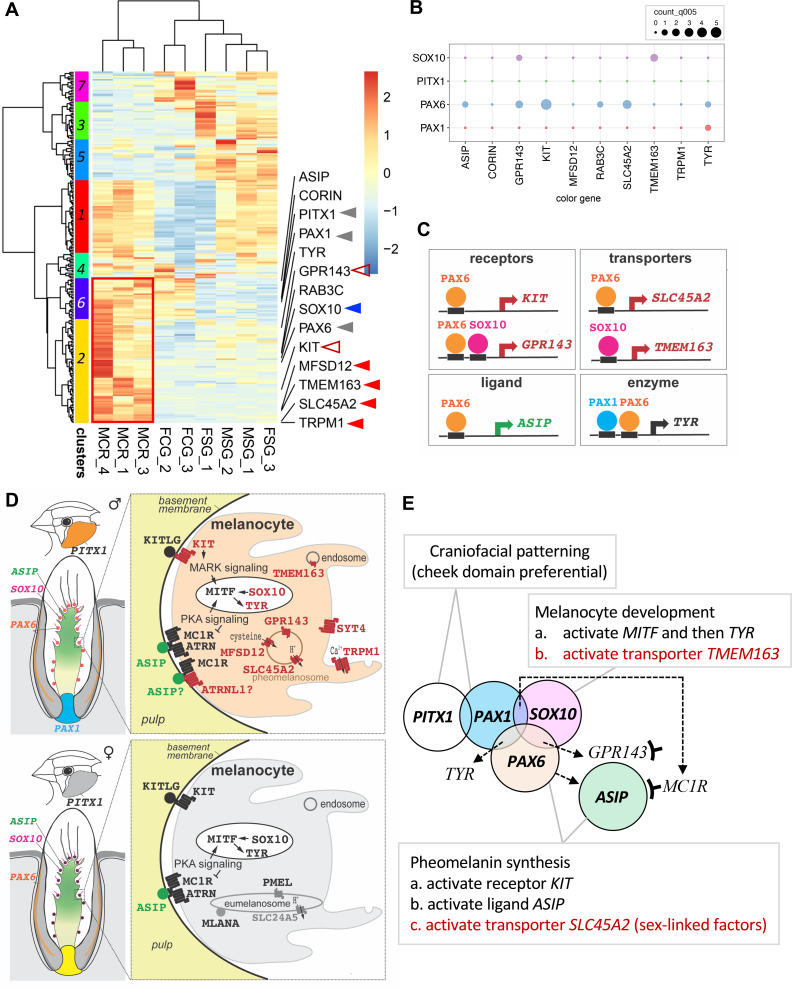
Potential regulatory hierarchy of regional TF genes, melanogenesis and sex-linked genes. (A) Hierarchical clustering heatmap derived from wild-type samples, indicating the co-expression between the three regional TF genes (gray arrows) and the eleven melanogenesis genes that are preferentially expressed in red cheek feathers within clusters 2 and 6. (B) Binding strength between four TFs (PITX1, PAX1, PAX6, and SOX10) and 10 melanogenesis genes. Binding strength was determined by counting TF binding to gene promoters and is illustrated using dot plots, where larger dots indicate higher binding strength. (C) Illustration outlining the predicted regulation of TF genes on the promotors of genes related to pheomelanin synthesis based on (B). (D) Schematic drawings of sex-dichromatic cheek feathers in male and female zebra finches, highlighting molecular candidates directing male-biased expression (red) in melanocytes of red cheek feathers. (E) A hypothetical regulatory hierarchy to connect cheek domain’s preferential TF profile with pigmentation pathway responsible for the enriched pheomelanin red color. The patterning of cheek domain is predicted to be related to PITX1 due to the detection of its transcripts within embryonic feather buds and adult feather sections from cheek domain. PAX1 in cheek dermal fibroblasts may serve as an upstream regulator, potentially triggering the male-biased color pattern through PAX6 and SOX10. PAX6 and SOX10 in melanocytes may enhance the expression of GPR143, SLC45A2, and TMEM163, driving increased pheomelanin production in males.

To elucidate potential connections between the cheek-regional TF genes and the pigmentation pathways involved in red cheeks, we conducted: (1) a gene co-expression analysis by generating a hierarchical cluster heatmap using the total DEGs (Fig 6A and S5 File) and (2) transcription factor binding analyses to predict their interactions with the color genes (Fig 6B). In this gene co-expression analysis, we included all the DEGs instead of only the color genes, as was done in Fig 2B, because we wanted to explore the regional transcription factors co-expressed with the color genes. The total DEGs were derived by merging all DEGs from the comparisons shown in Fig 2A. In the hierarchical cluster heatmap, we observed that three regional TF genes are clustered with eleven DEGs that are up-regulated in male red cheeks (clusters 2 and 6, Fig 6A). Among these eleven DEGs, SOX10 stands out as a critical TF involved in melanocyte development and activation of melanogenesis pathways (blue arrowhead, Fig 6A). The other ten DEGs include two melanocyte receptors (KIT and GRP143; open red triangles) and four transporters (SLC45A2, TRPM1, MFSD12, and TMEM163; closed red triangles) (Fig 6A), which are prominently expressed in the male red cheeks (S6B and S6D Fig) and play a pivotal role in facilitating pheomelanin synthesis.

In the second part of the analysis, we used the four TFs identified above (three regional and one male-red cheek enriched) to explore their interactions with the aforementioned eleven melanogenesis genes involved in red cheek coloring through transcription factor binding strength analysis (Fig 6B). Previous studies have shown that TF binding strength correlates with the number of binding site motifs for a particular TF found in bound regions [49]. Specifically, we calculated the number of binding site motifs for the four TF genes within genomic sequences spanning 2 kb upstream (including the putative promoters) and 1 kb downstream of the transcriptional start site of the ten co-expressed red cheek genes. We identified multiple binding motifs of the male-cheek-melanocyte-enriched PAX6 on the promoters of various genes, including the upstream regulators *KIT* and *ASIP*, as well as the downstream transporter *SLC45A2*, which facilitates pheomelanin synthesis (Fig 6B and 6C). SOX10 is likely to enhance the male-biased expression of *TMEM163*, encoded by *SLC30A11*, which has recently been identified as a zinc efflux protein [50], in the process of pheomelanin synthesis (Fig 6B). SOX10 may also collaborate with PAX6 to initiate the expression of *GPR143* (Fig 6B and 6C). Additionally, PAX1 interacts with *TYR*, with the potential involvement of PAX6 (Fig 6B and 6C). Despite PITX1 being recognized as a regional TF near the ear hole, we found no evidence of its interaction with the ten melanogenesis genes, suggesting that other intermediate factors could be involved in the regulation.

Based on the predicted and validated data, we summarize the regulatory hierarchy that contributes to the sex dichromatism of the craniofacial pattern in zebra finches in Fig 6D and 6E. The establishment of craniofacial patterning in the cheek domain involves the embryonic expression of *PITX1* in the cheek domain (S4E Fig). After that, two cheek-regional TF genes, PAX1 and PAX6, may activate the differential expressions of the ligand of ASIP, the enzyme TYR, the receptors KIT and GPR143, as well as the transporters SLC45A2, TMEM163, for pheomelanin synthesis (Figs 3 and 4). Notably, PAX6 appears to play a dual role in cheek domain specification and red cheek coloring (Figs 3E and 6C), although *ASIP* is up-regulated in both male and female cheek feathers (Fig 4).

### Comparative analyses of regional TF genes and melanogenesis genes across Passerine bird genomes

**Fig 7:**
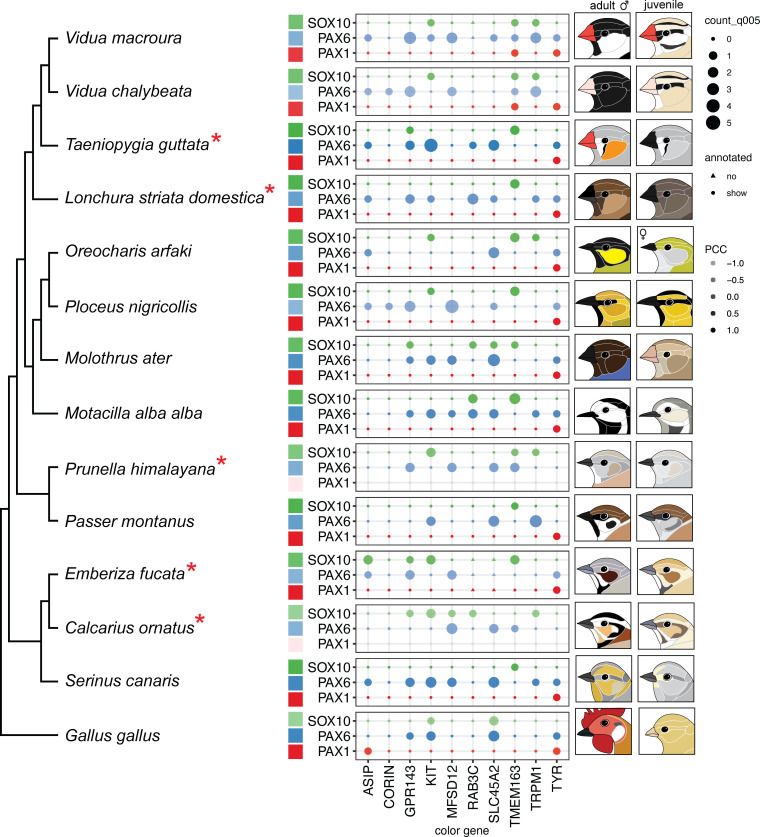
Binding strength between regional TFs and melanogenesis genes across passerines. Comparison of binding strength between the 3 regional TF genes (Y axis) and the 10 melanogenesis genes (X axis) across passerines, based on our working hypothesis (Fig 6B). The phylogenetic tree of the representative passerines is rooted by chickens. Schematic drawings depict craniofacial plumage of mature males and juvenile birds. The label “annotated” denotes the presence of melanogenesis genes in the genome annotation, while “count” signifies the counts of the transcription factor binding motifs in melanogenesis gene promoters. In each species, lower dot transparency, as seen in the society finch *Lonchura striata domestica* and Eurasian tree sparrow *Passer montanus*, indicates a higher Pearson correlation coefficient (PCC) in the overall TF-melanogenesis gene binding profile compared to that in the zebra finch *Taeniopygia guttata*. The higher Pearson correlation coefficient implies greater gene regulatory similarity between species. Red asterisks indicate bird species with distinct cheek domains exhibiting bright colors ranging from orange to brown, potentially influenced by pheomelanin pigments, similar to the cheek domain in zebra finches. The results suggest convergent evolution of cheek patterning.

The craniofacial plumage of passerine birds is characterized by the color pattern transition from juvenile to adulthood, leading to sexual dichromatism (Fig 1A). It is common to observe an enlarged colorful cheek domain that covers the ear holes in the passerines after sexual maturation (Fig 7). While these domains exhibit diverse shapes and colors across species, we suggest that the ear holes function as signaling centers for the development of auricular tracks, contributing to distinct finch cheek patterns. To explore the evolution of binding strength of the three predicted cheek regional TF genes and melanogenesis genes, we selected 13 passerine species spanning seven families (Estrildidae, Passeridae, Fringillidae, Motacillidae, Icteridae, Paramythiidae, and Ploceidae) based on the craniofacial plumage and the availability of their high-quality genome assemblies, using the chicken as the outgroup for our analysis (Fig 7). We depicted the craniofacial plumage of sex-dimorphic patterns from juveniles and adult males of these passerines (Fig 7).

To test whether the diversified cheek patterns resulted from convergent or parallel evolution, we examined the Pearson correlation coefficients of the TF-melanogenesis gene binding strength during finch evolution. Interestingly, the correlations are higher in closely related species, such as the society finch, *L. striata domestica*, than in passerine species exhibiting brighter cheek colors, similar to those of zebra finches (indicated by asterisks, Fig 7). This analysis suggests the possibility of convergent evolution of vivid cheek coloring, accompanied by distinct binding interactions between conserved regional signals and diversified melanogenesis genes. Moreover, the high correlations of the TF-color gene bindings between closely related species with opposite cheek brightness suggest that multiple pathways are involved in cheek color regulations (Fig 7).

## Discussion

In this study, we found that the red cheek patch in zebra finches is created through two layers of controls: first, the cheek domain is established by region-specific TFs during the embryonic development; second, the coloring is implemented during the secondary feather transition. Therefore, analyzing different developmental stages—embryonic, juvenile and mature stages—is crucial. Regarding the establishment of facial pigmentation domains, TFs such as PITX1 and PAX1 are expressed in the cheek domain, while HOX are expressed in the zebra finch scalp. Although no clear evidence has been found demonstrating the role of HOX in the scalp, *HOX* genes remain important for regional specificity [51]. Although the timing of when craniofacial dermal cells obtain their regional specificity from the neural crest and/or pharyngeal arches remains unresolved, the two-step coloring mechanism was also observed in the color pattern diversity in the trunk in estrildid finches, originating from somite, somatopleura and trunk neural crest cells in the previous findings [20]. Region-specific TF genes may interact with different color-implementation genes, opening doors for divergent colors in the same domain. With this principle established in two independent studies, it will be exciting to further explore the mechanisms in the future.

### Non-canonical ASIP-MC1R signaling and its implications for pigment regulation in zebra finch cheek variants

*ASIP* is expressed in the cheeks but does not lead to pheomelanin production in female gray cheeks, while increased eumelanin production is observed in black cheeks. This phenomenon is challenging to reconcile with the commonly described role of MC1R–ASIP interaction in mammals, where it regulates pheomelanin synthesis [52]. Thus, we suggest that some birds may utilize alternative mechanisms to regulate the switch of eumelanin and pheomelanin synthesis. Additional modifiers of the pigment-type switching pathway potentially may be involved. Previous studies have identified that ASIP–MC1R signaling includes a cAMP-independent pathway through ATRN (Attractin), a receptor located on melanocytes that acts as an ASIP accessory protein [53]. ATRN binds to the N-terminal of ASIP, increasing its affinity for MC1R [53]. In contrast, its paralog ATRNL1 (Attractin Like 1), interacts with the C terminus of MC4R [54].

Notably, although no differential expression of ATRN across zebra finch cheek color variants and sexes is observed, higher expression levels of ATRNL1 are detected in male red cheeks (S6E Fig and S1 and S4 Files). Therefore, it is possible that a non-canonical mechanism of ASIP-MC1R signaling, mediated by ATRNL1 proteins contributes to the male red cheeks in the zebra finches. Pheomelanin production in female gray cheek could be reduced due to inefficient ASIP-MC1R binding, resulting from low ATRNL1 levels, while eumelanin production would not be inhibited in black cheeks. How ATRNL1 contributes to the sexual dichromatism and cheek color variations in zebra finches deserves further investigation, particularly in the black cheek variant, which lacks a sex difference. In the case of black cheeks, the excess eumelanin might also result from constitutive activation mutations or recessive black mutations of MC1R, similar to those observed in turkeys [55].

### Sexual dichromatism of zebra finch can be contributed by genes located on the sex chromosomes

In avian species, positive selection underlying accelerated Z chromosome evolution has implicated the relaxed purifying selection or adaptive evolution in male gonad genes as contributing factors to sexual dichromatism [56,57]. Moreover, specific Z chromosome loci have been linked to speciation within the *Passerina* genus [58]. In zebra finches and chickens, 10–60% of male-biased Z-linked genes exhibit dosage effects in various tissues [59], despite the absence of a clear-cut dosage compensation mechanism in birds [60]. Z-linked genes have even been proposed to play a role in regulating sexually dimorphic zebra finch brain development and sexually dichromatic mallard craniofacial plumage [61,62].

In this study, we identified 18 Z-linked genes with strongly male-biased expression (absolute log2 fold change > 2) in red cheek feathers, which are influenced not only by dosage effects but also potentially by epigenetic regulation under hormonal effect. Further studies could explore the interplay between hormonal regulation and intrinsic cell-autonomous mechanisms linked to sex chromosome composition, such as epigenomic profiling of hormone-responsive elements using ChIP-seq for hormone receptors and examining the signals on the sex chromosomes. Among the 18 genes, *DMRT1* is well known for its role in sexually dimorphic patterns in animals [63]. However, its function in birds has so far only been identified in sex determination [64]. In *Drosophila*, male-specific pigmentation is a recent evolutionary innovation, controlled by homologs of *DMRT1* and *HOX* genes, *dsx* (*double-sex*) and *Abd-B* (*Abdominal-B*) [65]. Future work in zebra finches could focus on the *cis*-regulatory changes of *DMRT1* and its downstream targets.

### The convergent evolution of facial patterns in finches

Avian “faces” exhibit remarkably diverse color patterns, implying a cognitive function among species. Many of these colors are “painted” onto relatively conserved spatial domains, such as the crown (scalp), eyeline, cheek, and auricular regions, similar to those found in estrildid finches [11]. We hypothesize that this occurs by linking region-specific TF with different coloring mechanisms for optimal adaptation. In the exemplary comparative motif analyses (Fig 7), we found that diverse passerine facial colors are produced by conserved craniofacial skin domains (set up during the primary feather transition) and convergently evolved coloring mechanisms (set up during the secondary feather transition). Interestingly, although we focus on avian melanogenesis-related facial color evolution, our findings are similar to those for avian carotenoid-color evolution. Carotenoids, a trait that also evolved independently across birds, likely became prominent in bird coloration as avian lineages diversified alongside angiosperms during the Mesozoic time. Plants produce carotenoids in fruits and seeds to attract dispersers, and birds that exploited these resources gained access to these pigments. Over time, deposition of carotenoids in integumentary tissues may have shifted from a metabolic byproduct to a selected trait for signaling [66]. While melanogenesis-based coloration in birds is more likely to be a product of sexual selection, different selection pressures in various finch species may occasionally produce similar phenotypes.

In addition to colors, several important traits such as flight ability and super-black plumage, have convergently evolved multiple times [67–69]. Passerines are the most abundant bird lineage and underwent super-radiation to adapt to various eco-spaces [70,71]. While the conserved regional domains suggest that craniofacial regional specification evolves relatively slowly, color diversity in craniofacial feathers may evolve independently and rapidly through sexual selection [72].

### Future directions

In summary, craniofacial color patterns in birds are complex and play an important role in mating choices (Fig 7). While we cannot capture all the specific details across the many bird species, we can identify fundamental principles that guide the organization of these color patterns. From our analyses, two new insights emerge, which will help shape future research in this area. First, the red cheek color in male finches is known to be derived from pheomelanin, and we expected to find sex hormone-dependent expression of ASIP specifically in the male cheek [73,74]. However, our data suggest the alternative mechanisms for the red cheek patterning. Convergent selection stabilizes advantageous color traits derived from different biological pathways. The function of these pathways will be investigated in future studies, likely through genetic approaches.

Second, while the color patterns on birds’ faces may appear highly diverse, there is an underlying uniformity based on craniofacial integumentary domains. These topographic domains have been described anatomically (S2 Fig) [34]. In passerine birds, this basic structural design remains consistent; however, the topology (size and shape of each domain) varies among species and is ‘painted’ with different colors (e.g., cheek, eyeline, and scalp domains in Fig 7), resulting in the complex color patterns observed in nature. Future epigenomic studies will help clarify these mechanisms. Together, the findings from this study provide new insights, advancing our understanding of the multi-faceted processes involved in avian facial colors.

## Materials and methods

### Ethics statement

All the animals *T. guttata* used in this study were processed following the approved protocols of the Institutional Animal Care and Use Committees (IACUC 109–167) of the University of National Chung University (NCHU; Taichung, Taiwan).

### Animals

Nineteen zebra finches, which reach sexual maturity at the age of approximately 13 weeks and exhibit fully saturated red legs and beaks, were purchased from the local bird farm in Tainan, Taiwan. These included: (1) sixteen dominant wild-type phenotypes, referred as Normal Gray (NG), characterized by males with red cheek and females with gray cheeks; (2) two recessive mutant phenotypes, known as Black Cheek (BC), featuring black cheeks; and (3) two sex-linked mutant phenotypes, called Chestnut Flank White (CFW), characterized by light red cheeks (Figs 1 and S1). Among them, three pairs of wild-type finches were maintained to obtain fertilized eggs. The eggs were maintained at 38°C for hatching. Around 9 embryos at day 6–8 were staged [75] and collected to observe head feather bud growth and to perform whole-mount *in situ* hybridization. The juveniles were incubated at room temperature to monitor feather tract growth from post-hatching days 1–14.

### Biological specimen collection

To gather transcriptome profiles from different cheek colors, we plucked feathers from the anterior cheek domain and the center of the scalp domain on top of the head from thirteen finches (six NG males, three NG females, two CFW males, and two BC males). The morphology and the size of feathers from the cheek domain differ from the scalp domains (S1B Fig). Cheek feathers range in length from 4 to 8 mm, while scalp feathers are approximately 9 mm long. Additionally, red feathers from the anterior cheek domain are shorter and predominantly red, measuring approximately 4 mm in length, or display red coloring on two-thirds of the feather when the length is around 6 mm. In contrast, feathers from the posterior cheek domain exhibit red coloring on only one-half of the feather, with lengths ranging from 6 to 8mm. Based on these differences, we specifically chose to collect regenerated feathers from the anterior cheek domain for this study. Growing feathers from these domains were collected at an early stage, specifically within the one half of their growth, 7–26 days after plucking to account for the individual varying variation in growth rates. The collected growing feathers measured less than to 3 mm for cheek domain and 4.5 mm for scalp domain (S1C and S1D Fig).

### Paraffin section and immunofluorescent staining

Regenerating pigmented feather follicles were collected from two to three individuals for each color and fixed in 4% PFA at 4°C overnight, then serially dehydrated in alcohol, cleared in xylene and embedded in paraffin. Longitudinal sections were cut at a thickness of 7 μm for further immunofluorescent analysis. Antigen retrieval was performed at 98°C for 20 minutes. Primary antibodies used in this study include rabbit anti-K17 antibody (1:50, ab537097, Abcam), rabbit monoclonal anti-SATB1 (1:100, ab109122, Abcam), mouse anti-MiTF (C5) (1:100, ab12039, Abcam), rabbit anti-TRP1 (1:200, ab83774, Abcam), rabbit anti-SOX8 (1:200, ab27655, Abcam; cross reactivity against SOX10) [76], and mouse anti-PAX6 (1:50, Santa Cruz, sc-81649), which were incubated at 4°C overnight. The secondary antibodies, Alexa Fluor 488 goat anti-mouse IgG (H + L) (1:200, Cat #A-11001, Invitrogen), Alexa Fluor 647 anti-rabbit IgG (H + L) (1:200, Cat #A32733, Invitrogen), were used and incubated for 2 hours at room temperature. The antifade mounting medium (Fluoroshield with DAPI, Cat #F6057, Sigma-Aldrich) was added and fluorescent images were captured by a confocal microscope (Leica STELLARIS 8, Core Facility of Taipei Medical University).

### RNA sequencing

Ten to twelves growing feathers were used for RNA extraction for each sample. Each group had two to three replicates. Total RNAs were extracted followed by the standard Trizol extraction protocol and elute in 50 µl RNase-free water (Cat #15596026, Invitrogen). RNA purities and concentration ranging from 217 to 667 ng/ µl were accessed by the A260/A280 ratio determined by NanoDrop 2000 (Thermo Fisher Scientific) and RNA integrity was calculated by comparing 28S and 18S intensity through capillary electrophoresis (Agilent BioAnalyzer and Agilent TapeStation). RNA-seq libraries were prepared using TruSeq RNA Prep Kit v2 (Cat #RS-122–2001/-2002, Illumina). 1 µg of total RNA was used to prepare one cDNA library and oligo-dT affinity beads in the kit were used to purify mRNA and other RNAs containing poly-A sequences. After library amplification, clean-up was performed for size selection and removal of adapter-dimers by 0.8X AMPure XP bead (Cat #A63880, Beckman) according to the previous study [77]. Sequencing was performed by the USC Molecular Genomic Core (MGC) on the NextSeq500 platform using the single-end 75-bp protocol (Illumina).

### RNA-Seq data analysis

Low-quality reads were removed using Trimomatic [78] according to the following procedure: 1) Remove adaptors; 2) remove leading low-quality bases below Q score 18; 3) remove trailing low quality bases below Q score 18; 4) remove average Q score below 15 in 4 bp sliding window, and 5) drop trimmed reads below 36 bases long. The zebra finch genome assembly (bTaeGut1.4.pri) and its annotation were downloaded from NCBI Reference Sequence (RefSeq) database [79]. The processed sequencing reads were mapped to the genome using HISAT2 with default parameters, resulting in an average of 39.3M reads per sample with a standard deviation of 5.3M reads (S4 File) [80]. The read counts and the Transcripts Per Kilobase Million (TPM) values for each gene were obtained from the mapping files using StringTie with -e and -B parameters [81,82]. Only protein-coding genes were retained for the follow-up analyses. Upper quartile normalization was applied to the TPM table (S4 File). To exclude lowly expressed genes, genes with the mean TPM value greater than 1 across libraries were defined as the expressed genes.

### Identification of differentially expressed genes (DEGs) and construction of the heatmaps

Read counts of the genes across the libraries were normalized using the median of ratios method, and the DEGs were calculated using DESeq2 [83]. The raw read counts of the genes were performed regularized log transformation by DESeq2 [83] and the transformed values (rld) were used in generating hierarchically clusters and heatmaps by the R package ‘pheatmap’ [84]. Ward.D2 was used as the agglomeration method.

### Reverse transcription and quantitative PCR (qPCR)

qPCR was performed using two biological replicates and two technique replicates. For reverse transcription (RT), Oligo (dT)20 (Cat #18418020, Thermo Fisher), dNTP Mix (Cat #R0192, Thermo Fisher), and ThermoScript Reverse Tran‑scriptase (Cat #12236–014, Invitrogen) were used. Each RT reaction (20µl) utilized a fixed amount of total RNA (1µg). Each cDNA sample was diluted to 1/4X, and 1µl of the 1/4X diluted cDNA was used for qPCR reaction (20µl). qPCR was conducted using Maxima SYBR Green/ROX qPCR Master Mix (2x) (Cat #K0222, Thermo Fisher). qPCR primers for *PAX1* are 5’-GGCGACATCAAATACCCCCAG-3’ and 5’-TTTCTGTCCACCCCTTCACCG-3’ (reverse); for *PAX6* are 5’-AGAGGGGGTCTGTACCAACG-3’ and 5’-CCCGTTCAGCATCCTTAGCTT-3’ (reverse); for *PITX1* are 5’-ACCCGGCGAAGAAGAAGAAG-3’ and 5’-ACAGGTCCATCTGCTGGTTC-3’ (reverse); for *ASIP* are 5’-AAAATCCCAGAAAGTCAGCAG-3’ and 5’-TGTGGCTTGCAGGTTTTGAAG-3’ (reverse); for *MC1R* are 5’- CTCATCGTCACCTGTCCCAC-3’ and 5’-ATGAGGGGGTCAATCACCGA-3’ (reverse); for *MITF* are 5’-TGCTCAGAATACAGGAACTCGAGA-3’ and 5’- TAGGTGCCAGCTGGCTCAGT-3’ (reverse); for *SOX10* are 5’- CGGAACACTCCTCAGGTCAGA-3’ and 5’-TTGGACATCACCTCATGGCT-3’ (reverse); for *SLC45A2* are 5’-GAGTCATGTCCAGCACGCTA-3’ and 5’- CCTGCTTGTTTCCCCTTCAATAC-3’ (reverse); for reference gene GAPDH are 5’-TTGTCAGCAATGCTTCCTGCA-3’ and 5’- GCAGCACCTCTGCCATC-3’ (reverse). Every primer pair was serial diluted to 1X, 5X, 25X, and 125X, and examined in qPCR to obtain its regression equation, qPCR primer efficiency, and amplification factor (E). The primer efficiency is located between 93.6 to 108.9%. The melting curve analysis was checked for each primer pair to confirm the presence of a single major peak at melting temperature (Tm) and the absence of additional peaks below Tm, indicating no primer dimers in the reaction. The relative gene expression ratio was calculated according to the Pfaffl formula. The Pfaffl formula is: Gene expression ratio = [(E of gene of interest)^∆Ct of gene of interest]/[E of reference gene)^∆Ct of reference gene]. E is amplification factor, calculated as E = 10^(−1/ slope of primer regression equation).

### Whole-mount *in situ* hybridization (WISH) on zebra finch embryos

Zebra finch embryos were collected and fixed in 4% paraformaldehyde at 4°C overnight. For whole-mount ISH, embryos were dehydrated through a methanol series and followed the procedures described by [85]. For WISH probes gene-specific fragments were amplified from total RNA extracted from entire E6 zebra finch embryos and subsequently cloned into pDrive cloning vector system (Cat #231124, Qiagen). The following prime pair was used to clone zebra finch *PITX1* (nucleotides 501–1256; XM_004175411.4): forward: CCAGAGACCCGATCGACAAC; reverse: ACTGCTGTAGCCGAAACTGG. Digoxigenin-labeled RNA probes were made by *in vitro* transcription according to the instructions from the manufacturer (Cat #11175025910, Roche).

### RNAscope fluorescent *in situ* hybridization

Fluorescent *in situ* hybridization was performed using RNAscope Multiplex Fluorescent Reagent Kit v2 according to the manufacturer’s protocol (Cat #323100, ACDBio). Briefly, tissue sections were baked for 1 h at 65°C, deparaffinized, and treated with H_2_O_2_ for 10 min at room temperature. Target retrieval was performed for 15 min at 100°C, followed by protease treatment for 30 min at 40°C. Zebra finch specific RNAscope Probes for *ASIP*, *PAX1* and *PITX1* genes were synthesized (Tgu-ASIP-C1, Cat #1151081-C1; Tgu-PITX1-C2, Cat #1151101-C2; Tgu-PAX1-C1, Cat #1204171-C1; ACD). Probe C2 was diluted in probes C1 (1:50) and hybridized for 2 h at 40°C to detect the expressions of on longitudinal sections of zebra finch feather follicles. Probes were then followed by RNAscope amplification and using Optal dye 690 and 520 (1:750 dilution in TSA buffer) to show the fluorescent signals.

### Evolutionary analysis of the transcription factor binding strength

To analyze the evolution of the binding affinities between the co-expressed melanogenesis genes and regional transcription factors predicted in this study, we selected the genomes of several finch species with colored cheeks based on the following assembly criteria: scaffold N50 greater than 500 kb and the number of scaffolds fewer than 30,000. *Calcarius ornatus* (GCA_013397715.1), *Emberiza fucata* (GCA_013398835.1), *Gallus gallus* (GCF_016700215.2), *Lonchura striata* (GCF_005870125.1), *Molothrus ater* (GCF_012460135.1), *Motacilla alba* (GCF_015832195.1), *Oreocharis arfaki* (GCA_013398015.1), *Passer montanus* (GCF_014805655.1), *Ploceus nigricollis* (GCA_013399945.1), *Prunella himalayana* (GCA_013398875.1), *Serinus canaria* (GCF_022539315.1), *Vidua chalybeata* (GCA_013398495.1), and *Vidua macroura* (GCF_024509145.1) were selected for the analysis. For each species, genomic sequences spanning 2 kb upstream to 1 kb downstream of the transcriptional start site for each color gene were extracted as the putative promoter sequences. All promoters of the three annotated *ASIP* gene isoforms were included based on this criterion. DNA‐binding motif analyses were performed on these promoter sequences using AnimalTFDB 3.0 [86]. We tested the binding strength between the co-expressed melanogenesis genes and regional TFs based on two criteria: 1. The false discovery rates of the binding scores between the promoters of the co-expressed melanogenesis genes and regional TFs were lower than 0.05 (padj < 0.05). 2. A greater number of binding hits between the promoter and the TFs was defined as higher binding strength. The avian phylogeny was obtained from NCBI, which is curated by taxonomists based on the current consensus in scientific literature [87], and visualized using MEGA software [88].

### Statistical analysis

In the DEG analysis, genes were defined as differentially expressed if their log2 Fold Change was greater than 1 or less than −1, and the false discovery rate (padj, the Wald test) was less than 0.05 between comparisons (S1 File). To compare the DEG numbers between autosomes and sex chromosomes, a permutation test for linear models was applied using the R package *lmPerm* (Fig 5A) [89]. To confirm the samplings and DEG analysis, the g:GOSt program embedded in g:Profiler, a public web server for characterizing and manipulating gene lists resulting from mining high-throughput genomic data, was applied to each DEG set. The mouse database was used to increase the sensitivity of the analysis, and the Benjamini-Hochberg FDR threshold was set to less than 0.01 (S1 File). To quantify the binding affinities between the co-expressed melanogenesis genes and regional transcription factors, we calculated and plotted the PCC (Pearson correlation coefficient) values based on the hit number between the regional transcription factors and the promoters of the melanogenesis genes across the finches.

## Supporting information

S1 Fig Temporal development of feathers in craniofacial domains and the collection of regenerated feathers from wild-type and mutant zebra finches. (A) Depiction of the progression of feather growth on juvenile heads from post-hatching day1–10 (D1 to D10). Arrowheads indicate the emerging feather follicles beneath or above the skin. (B) A comparison of the distinct morphology and color between the cheek versus the scalp feathers (the top of head, also referred to as the crown) [34]. Notably, the scalp feather exhibits longer barbs as compared to the cheek feather. (C, D) The strategy for collecting regenerated feathers from the cheek domain (C) and the scalp domain (S) of zebra finches includes three basic types of genetic traits: dominant, recessive, and sex-linked. The dominant wild-type phenotype is called Normal Gray (NG), characterized by males with red cheek and females with gray cheeks. The recessive mutant phenotype, featuring black cheeks, is called Black Cheek (BC), while the sex-linked mutant phenotype, characterized by light red cheeks, is called Chestnut Flank White (CFW).
(PDF)

S2 Fig Topography of feather tracts on head domains. (A) Single Comb White Leghorn chicken *Gallus gallus*. (A’) Auricular tract of Single Comb White Leghorn chicken. (B) Common pigeon. These images are adapted from Figures 53, 55, and 59 in Chapter 3 of the book Avian Anatomy: Integument, 1972. U.S. Agricultural Research Service; U.S. Govt. Print. Off. https://www.google.com.tw/books/edition/Avian_Anatomy_Integument/phJDrhR7RS8C?hl [34].
(PDF)

S3 Fig Volcano plots illustrating different comparison groups in transcriptome. The comparisons between different domains (scalp and cheek) are presented in (A, B), between cheek feather colors is displayed in (C–F), between sexes is shown in (G), and between male cheek red (MCR) feathers and others is exhibited in (H). Genes exhibiting statistically significant changes (padj < 0.05, log2 Fold Change > 1 or <-1) are marked as red (log2 Fold Change > 1) or blue dots (log2 Fold Change < -1), respectively. The number of differentially expressed genes (DEGs) in each panel is indicated in the upper left or right corner. The significant DEGs for each comparison are listed in S1 File.
(PDF)

S4 Fig Expression profiles of other scalp- or cheek enriched genes. (A) Expression of other representative *HOX* genes exhibiting high TPM levels in scalps. (B) qPCR validation of genes with high TPM levels in cheeks as shown in Fig 3C. (C) Immunohistochemistry using SATB1 antibody. SATB1 proteins are predominantly expressed in the nuclei of keratinocyte (KT) within scalp feathers as opposed to cheek feathers. (D) RNAscope analysis utilizing *PITX1* probes shows that *PITX1* mRNA is randomly distributed within cheek feathers, while no *PITX1* mRNA is detected in scalp feathers. (E) Whole-mount *in situ* hybridization employing *PITX1* probes on finch embryonic day 7 and 8 (E7 and E8). Preferential expression of *PITX1* is observed within developing feather buds on the cheek (see magnified panels below) and eyebrow regions from E8 (arrowheads), compared to scalp region (hollow arrowheads). Abbreviation: er, ear hole; ot, otic vesicle.
(PDF)

S5 Fig Expression of melanogenesis genes in melanocytes of cheek feathers. (A) TRP1 proteins are detected in the cytoplasm of certain melanocytes engaged in eumelanin synthesis. (B) Similar numbers and distributions of melanocyte nuclei, which were detected by MITF antibody, within male and female cheek feathers. (C) qPCR validation of *ASIP*, *MC1R*, *MITF*, and *SOX10* genes. (D) SOX10 proteins are expressed in melanocytes and partial keratinocytes of both male and female cheeks. Abbreviation: KT, keratinocytes; M, melanocytes.
(PDF)

S6 Fig Distribution of DEGs across chromosomes and representative DEGs highly expressed in male cheek red feathers. (A) Distribution of DEGs from the cheek comparison (orange dots, MCR vs. FCG) and scalp comparison (gray dots, MSG vs. FSG) across chromosomes. (B–E) TPM levels of genes encoding transporters (B), *MLANA* (C), receptors (D), and *ATRN* and *ATRNL1* (E).
(PDF)

S1 File Statistics and gene enrichment analysis of the DEGs from different comparisons in Fig S3. (XLSX)

S2 File List of genes related to melanogenesis and carotenoid pathways in animals for Fig 2. (XLSX)

S3 File Gene list of each hierarchical clustering from all DEGs for Fig 2B. (CSV)

S4 File RNAseq summary, TPM table and Ensembl IDs. (XLSX)

S5 File Gene list of each hierarchical clustering from all DEGs in Fig 6A. (CSV)

## References

[1] Ramos R, Swedlund B, Ganesan AK, Morsut L, Maini PK, Monuki ES, et al. Parsing patterns: Emerging roles of tissue self-organization in health and disease. Cell. 2024;187(13):3165–86. doi: 10.1016/j.cell.2024.05.016 38906093 PMC11299420

[2] Gomes ACR, Funghi C, Soma M, Sorenson MD, Cardoso GC. Multimodal signalling in estrildid finches: song, dance and colour are associated with different ecological and life-history traits. J Evol Biol. 2017;30(7):1336–46. doi: 10.1111/jeb.13102 28434197

[3] Wu P, Jiang T-X, Lei M, Chen C-K, Hsieh Li S-M, Widelitz RB, et al. Cyclic growth of dermal papilla and regeneration of follicular mesenchymal components during feather cycling. Development. 2021;148(18):dev198671. doi: 10.1242/dev.198671 34344024 PMC10656464

[4] Chuong C-M, Yeh C-Y, Jiang T-X, Widelitz R. Module-based complexity formation: periodic patterning in feathers and hairs. Wiley Interdiscip Rev Dev Biol. 2013;2(1):97–112. doi: 10.1002/wdev.74 23539312 PMC3607644

[5] Chen C-K, Chang Y-M, Jiang T-X, Yue Z, Liu T-Y, Lu J, et al. Conserved regulatory switches for the transition from natal down to juvenile feather in birds. Nat Commun. 2024;15(1):4174. doi: 10.1038/s41467-024-48303-3 38755126 PMC11099144

[6] Töpfer T. Morphological Variation in Birds: Plasticity, Adaptation, and Speciation. In: Tietze DT, editor. Bird Species: How They Arise, Modify and Vanish. Cham: Springer International Publishing; 2018, 63–74.

[7] Wright AE, Fumagalli M, Cooney CR, Bloch NI, Vieira FG, Buechel SD, et al. Male-biased gene expression resolves sexual conflict through the evolution of sex-specific genetic architecture. Evol Lett. 2018;2(2):52–61. doi: 10.1002/evl3.39 30283664 PMC6089503

[8] Bear A, Monteiro A. Both cell-autonomous mechanisms and hormones contribute to sexual development in vertebrates and insects. Bioessays. 2013;35(8):725–32. doi: 10.1002/bies.201300009 23804281

[9] Gazda MA, Araújo PM, Lopes RJ, Toomey MB, Andrade P, Afonso S, et al. A genetic mechanism for sexual dichromatism in birds. Science. 2020;368(6496):1270–4. doi: 10.1126/science.aba0803 32527835

[10] Kimball RT, Ligon JD. Evolution of Avian Plumage Dichromatism from a Proximate Perspective. The American Naturalist. 1999;154(2):182–93. doi: 10.1086/303228

[11] Zhao D, McBride D, Nandi S, McQueen HA, McGrew MJ, Hocking PM, et al. Somatic sex identity is cell autonomous in the chicken. Nature. 2010;464(7286):237–42. doi: 10.1038/nature08852 20220842 PMC3925877

[12] Lin SJ, Foley J, Jiang TX, Yeh CY, Wu P, Foley A, et al. Topology of feather melanocyte progenitor niche allows complex pigment patterns to emerge. Science. 2013;340(6139):1442–5. doi: 10.1126/science.1230374 23618762 PMC4144997

[13] Widelitz RB, Lin G-W, Lai Y-C, Mayer JA, Tang P-C, Cheng H-C, et al. Morpho-regulation in diverse chicken feather formation: Integrating branching modules and sex hormone-dependent morpho-regulatory modules. Dev Growth Differ. 2019;61(1):124–38. doi: 10.1111/dgd.12584 30569461 PMC6333526

[14] Arnold AP. The effects of castration and androgen replacement on song, courtship, and aggression in zebra finches (*Poephila guttata*). J Exp Zool. 1975;191(3):309–26. doi: 10.1002/jez.1401910302 1092802

[15] Leader N, Nottebohm F. Delayed plumage maturation in socially isolated juvenile zebra finches, *Taeniopygia guttata*. Animal Behaviour. 2006;72(1):113–21. doi: 10.1016/j.anbehav.2005.09.013

[16] Zann RA, Bamford M. The zebra finch: A synthesis of field and laboratory studies. Oxford University Press; 1996.

[17] Agate RJ, Grisham W, Wade J, Mann S, Wingfield J, Schanen C, et al. Neural, not gonadal, origin of brain sex differences in a gynandromorphic finch. Proc Natl Acad Sci U S A. 2003;100(8):4873–8. doi: 10.1073/pnas.0636925100 12672961 PMC153648

[18] Curantz C, Manceau M. Trends and variation in vertebrate patterns as outcomes of self-organization. Curr Opin Genet Dev. 2021;69:147–53. doi: 10.1016/j.gde.2021.05.001 34058514

[19] Deline B, Greenwood JM, Clark JW, Puttick MN, Peterson KJ, Donoghue PCJ. Evolution of metazoan morphological disparity. Proc Natl Acad Sci U S A. 2018;115(38):E8909–18. doi: 10.1073/pnas.1810575115 30181261 PMC6156614

[20] Haupaix N, Curantz C, Bailleul R, Beck S, Robic A, Manceau M. The periodic coloration in birds forms through a prepattern of somite origin. Science. 2018;361(6408):eaar4777. doi: 10.1126/science.aar4777 30237324

[21] Inaba M, Jiang T-X, Liang Y-C, Tsai S, Lai Y-C, Widelitz RB, et al. Instructive role of melanocytes during pigment pattern formation of the avian skin. Proc Natl Acad Sci U S A. 2019;116(14):6884–90. doi: 10.1073/pnas.1816107116 30886106 PMC6452743

[22] Hidalgo M, Curantz C, Quenech’Du N, Neguer J, Beck S, Mohammad A, et al. A conserved molecular template underlies color pattern diversity in estrildid finches. Sci Adv. 2022;8(35):eabm5800. doi: 10.1126/sciadv.abm5800 36044564 PMC9432839

[23] Price-Waldman RM, Shultz AJ, Burns KJ. Speciation rates are correlated with changes in plumage color complexity in the largest family of songbirds. Evolution. 2020;74(6):1155–69. doi: 10.1111/evo.13982 32333393

[24] Doucet SM, Mennill DJ, Hill GE. The Evolution of Signal Design in Manakin Plumage Ornaments. Am Nat. 2007;169(S1):S62–80. doi: 10.1086/510162 29517930

[25] Gomez D, Théry M. Simultaneous Crypsis and Conspicuousness in Color Patterns: Comparative Analysis of a Neotropical Rainforest Bird Community. Am Nat. 2007;169(S1):S42–61. doi: 10.1086/510138 29517929

[26] Delhey K. Revealing the colourful side of birds: spatial distribution of conspicuous plumage colours on the body of Australian birds. Journal of Avian Biology. 2020;51(1). doi: 10.1111/jav.02222

[27] Burley N, Coopersmith C. Bill color preferences of zebra finches. Ethology. 1987;76(2):133–51.

[28] Roberts ML, Buchanan KL, Bennett ATD, Evans MR. Mate choice in zebra finches: does corticosterone play a role?. Animal Behaviour. 2007;74(4):921–9. doi: 10.1016/j.anbehav.2006.12.021

[29] Templeton JJ, McCracken BG, Sher M, Mountjoy DJ. An eye for beauty: lateralized visual stimulation of courtship behavior and mate preferences in male zebra finches, *Taeniopygia guttata*. Behav Processes. 2014;102:33–9. doi: 10.1016/j.beproc.2013.11.001 24239504

[30] Shawkey MD, D’Alba L. Interactions between colour-producing mechanisms and their effects on the integumentary colour palette. Philos Trans R Soc Lond B Biol Sci. 2017;372(1724):20160536. doi: 10.1098/rstb.2016.0536 28533449 PMC5444072

[31] Mcgraw KJ, Wakamatsu K. Melanin Basis of Ornamental Feather Colors in Male Zebra Finches. The Condor. 2004;106(3):686–90. doi: 10.1093/condor/106.3.686

[32] Mundy NI, Stapley J, Bennison C, Tucker R, Twyman H, Kim K-W, et al. Red Carotenoid Coloration in the Zebra Finch Is Controlled by a Cytochrome P450 Gene Cluster. Curr Biol. 2016;26(11):1435–40. doi: 10.1016/j.cub.2016.04.047 27212402

[33] Fessing MY, Mardaryev AN, Gdula MR, Sharov AA, Sharova TY, Rapisarda V, et al. p63 regulates Satb1 to control tissue-specific chromatin remodeling during development of the epidermis. J Cell Biol. 2011;194(6):825–39. doi: 10.1083/jcb.201101148 21930775 PMC3207288

[34] Lucas AM, Stettenheim PR. Avian anatomy: integument. U.S. Agricultural Research Service; U.S. Govt. Print. Off.; 1972.

[35] Hoffman JI, Krause ET, Lehmann K, Krüger O. MC1R genotype and plumage colouration in the zebra finch (*Taeniopygia guttata*): population structure generates artefactual associations. PLoS One. 2014;9(1):e86519. doi: 10.1371/journal.pone.0086519 24489736 PMC3906038

[36] Domyan ET, Hardy J, Wright T, Frazer C, Daniels J, Kirkpatrick J, et al. SOX10 regulates multiple genes to direct eumelanin versus pheomelanin production in domestic rock pigeon. Pigment Cell Melanoma Res. 2019;32(5):634–42. doi: 10.1111/pcmr.12778 30838786 PMC6850303

[37] Fufa TD, Harris ML, Watkins-Chow DE, Levy D, Gorkin DU, Gildea DE, et al. Genomic analysis reveals distinct mechanisms and functional classes of SOX10-regulated genes in melanocytes. Hum Mol Genet. 2015;24(19):5433–50. doi: 10.1093/hmg/ddv267 26206884 PMC4572067

[38] Jia Q, Hu S, Jiao D, Li X, Qi S, Fan R. Synaptotagmin-4 promotes dendrite extension and melanogenesis in alpaca melanocytes by regulating Ca^2+^ influx via TRPM1 channels. Cell Biochem Funct. 2020;38(3):275–82. doi: 10.1002/cbf.3465 31743468 PMC7318172

[39] Yoo JC, Lim T yeon, Park JS, Hah Y-S, Park N, Hong S-G, et al. SYT14L, especially its C2 domain, is involved in regulating melanocyte differentiation. J Dermatol Sci. 2013;72(3):246–51. doi: 10.1016/j.jdermsci.2013.07.010 23999003

[40] Oancea E, Vriens J, Brauchi S, Jun J, Splawski I, Clapham DE. TRPM1 forms ion channels associated with melanin content in melanocytes. Sci Signal. 2009;2(70):ra21. doi: 10.1126/scisignal.2000146 19436059 PMC4086358

[41] De Mazière AM, Muehlethaler K, van Donselaar E, Salvi S, Davoust J, Cerottini J-C, et al. The melanocytic protein Melan-A/MART-1 has a subcellular localization distinct from typical melanosomal proteins. Traffic. 2002;3(9):678–93. doi: 10.1034/j.1600-0854.2002.30909.x 12191019

[42] Hoashi T, Watabe H, Muller J, Yamaguchi Y, Vieira WD, Hearing VJ. MART-1 is required for the function of the melanosomal matrix protein PMEL17/GP100 and the maturation of melanosomes. J Biol Chem. 2005;280(14):14006–16. doi: 10.1074/jbc.M413692200 15695812

[43] Giordano F, Bonetti C, Surace EM, Marigo V, Raposo G. The ocular albinism type 1 (OA1) G-protein-coupled receptor functions with MART-1 at early stages of melanogenesis to control melanosome identity and composition. Hum Mol Genet. 2009;18(23):4530–45. doi: 10.1093/hmg/ddp415 19717472

[44] Le L, Escobar IE, Ho T, Lefkovith AJ, Latteri E, Haltaufderhyde KD, et al. SLC45A2 protein stability and regulation of melanosome pH determine melanocyte pigmentation. Mol Biol Cell. 2020;31(24):2687–702. doi: 10.1091/mbc.E20-03-0200 32966160 PMC7927184

[45] Campagna L, Repenning M, Silveira LF, Fontana CS, Tubaro PL, Lovette IJ. Repeated divergent selection on pigmentation genes in a rapid finch radiation. Sci Adv. 2017;3(5):e1602404. doi: 10.1126/sciadv.1602404 28560331 PMC5443641

[46] Gunnarsson U, Hellström AR, Tixier-Boichard M, Minvielle F, Bed’hom B, Ito S, et al. Mutations in SLC45A2 cause plumage color variation in chicken and Japanese quail. Genetics. 2007;175(2):867–77. doi: 10.1534/genetics.106.063107 17151254 PMC1800597

[47] Abdel-Malek ZA. Fueling Melanocytes with ATP from Keratinocytes Accelerates Melanin Synthesis. J Invest Dermatol. 2019;139(7):1424–6. doi: 10.1016/j.jid.2019.03.1137 31230638

[48] Lee D, Takayama S, Goldberg AL. ZFAND5/ZNF216 is an activator of the 26S proteasome that stimulates overall protein degradation. Proc Natl Acad Sci U S A. 2018;115(41):E9550–9. doi: 10.1073/pnas.1809934115 30254168 PMC6187164

[49] Li X, MacArthur S, Bourgon R, Nix D, Pollard DA, Iyer VN, et al. Transcription factors bind thousands of active and inactive regions in the *Drosophila* blastoderm. PLoS Biol. 2008;6(2):e27. doi: 10.1371/journal.pbio.0060027 18271625 PMC2235902

[50] Styrpejko DJ, Cuajungco MP. Transmembrane 163 (TMEM163) Protein: A New Member of the Zinc Efflux Transporter Family. Biomedicines. 2021;9(2):220. doi: 10.3390/biomedicines9020220 33670071 PMC7926707

[51] Li J, Lee M-O, Davis BW, Wu P, Hsieh Li S-M, Chuong C-M, et al. The crest phenotype in domestic chicken is caused by a 197 bp duplication in the intron of *HOXC10*. G3 (Bethesda). 2021;11(2):jkaa048. doi: 10.1093/g3journal/jkaa048 33704432 PMC8022956

[52] Walker WP, Gunn TM. Shades of meaning: the pigment-type switching system as a tool for discovery. Pigment Cell Melanoma Res. 2010;23(4):485–95. doi: 10.1111/j.1755-148X.2010.00721.x 20465596

[53] Hida T, Wakamatsu K, Sviderskaya EV, Donkin AJ, Montoliu L, Lynn Lamoreux M, et al. Agouti protein, mahogunin, and attractin in pheomelanogenesis and melanoblast-like alteration of melanocytes: a cAMP-independent pathway. Pigment Cell Melanoma Res. 2009;22(5):623–34. doi: 10.1111/j.1755-148X.2009.00582.x 19493315 PMC2784899

[54] Haqq AM, René P, Kishi T, Khong K, Lee CE, Liu H, et al. Characterization of a novel binding partner of the melanocortin-4 receptor: attractin-like protein. Biochem J. 2003;376(Pt 3):595–605. doi: 10.1042/BJ20031241 14531729 PMC1223823

[55] Vidal O, Viñas J, Pla C. Variability of the melanocortin 1 receptor (MC1R) gene explains the segregation of the bronze locus in turkey (*Meleagris gallopavo*). Poult Sci. 2010;89(8):1599–602. doi: 10.3382/ps.2010-00726 20634512

[56] Dean R, Harrison PW, Wright AE, Zimmer F, Mank JE. Positive Selection Underlies Faster-Z Evolution of Gene Expression in Birds. Mol Biol Evol. 2015;32(10):2646–56. doi: 10.1093/molbev/msv138 26067773 PMC4576705

[57] Dutoit L, Mugal CF, Bolívar P, Wang M, Nadachowska-Brzyska K, Smeds L, et al. Sex-biased gene expression, sexual antagonism and levels of genetic diversity in the collared flycatcher (*Ficedula albicollis*) genome. Mol Ecol. 2018;27(18):3572–81. doi: 10.1111/mec.14789 30055065

[58] Carling MD, Brumfield RT. Speciation in Passerina buntings: introgression patterns of sex-linked loci identify a candidate gene region for reproductive isolation. Mol Ecol. 2009;18(5):834–47. doi: 10.1111/j.1365-294X.2008.04038.x 19207259

[59] Itoh Y, Melamed E, Yang X, Kampf K, Wang S, Yehya N, et al. Dosage compensation is less effective in birds than in mammals. J Biol. 2007;6(1):2. doi: 10.1186/jbiol53 17352797 PMC2373894

[60] Alekseyenko AA, Demakova OV, Belyaeva ES, Makarevich GF, Kotlikova IV, Nöthiger R, et al. Dosage compensation and intercalary heterochromatin in X chromosomes of *Drosophila melanogaster*. Chromosoma. 2002;111(2):106–13. doi: 10.1007/s00412-002-0191-7 12111333

[61] Chen X, Agate RJ, Itoh Y, Arnold AP. Sexually dimorphic expression of trkB, a Z-linked gene, in early posthatch zebra finch brain. Proc Natl Acad Sci U S A. 2005;102(21):7730–5. doi: 10.1073/pnas.0408350102 15894627 PMC1140405

[62] Ma S, Liu H, Wang J, Wang L, Xi Y, Liu Y, et al. Transcriptome Analysis Reveals Genes Associated With Sexual Dichromatism of Head Feather Color in Mallard. Front Genet. 2021;12:627974. doi: 10.3389/fgene.2021.627974 34956302 PMC8692775

[63] Kopp A. *Dmrt* genes in the development and evolution of sexual dimorphism. Trends Genet. 2012;28(4):175–84. doi: 10.1016/j.tig.2012.02.002 22425532 PMC3350790

[64] Smith CA, Roeszler KN, Ohnesorg T, Cummins DM, Farlie PG, Doran TJ, et al. The avian Z-linked gene DMRT1 is required for male sex determination in the chicken. Nature. 2009;461(7261):267–71. doi: 10.1038/nature08298 19710650

[65] Williams TM, Selegue JE, Werner T, Gompel N, Kopp A, Carroll SB. The regulation and evolution of a genetic switch controlling sexually dimorphic traits in *Drosophila*. Cell. 2008;134(4):610–23. doi: 10.1016/j.cell.2008.06.052 18724934 PMC2597198

[66] Geoffrey E, Kevin J. Bird Coloration. Geoffrey EH, Kevin JM, editors. Cambridge, MA and London, England: Harvard University Press; 2006.

[67] Harshman J, Braun EL, Braun MJ, Huddleston CJ, Bowie RCK, Chojnowski JL, et al. Phylogenomic evidence for multiple losses of flight in ratite birds. Proc Natl Acad Sci U S A. 2008;105(36):13462–7. doi: 10.1073/pnas.0803242105 18765814 PMC2533212

[68] McCoy DE, Prum RO. Convergent evolution of super black plumage near bright color in 15 bird families. J Exp Biol. 2019;222(Pt 18). doi: 10.1242/jeb.208140 31558610

[69] Terrill RS, Shultz AJ. Feather function and the evolution of birds. Biol Rev Camb Philos Soc. 2023;98(2):540–566. doi: 10.1111/brv.12918 36424880

[70] Barker FK, Cibois A, Schikler P, Feinstein J, Cracraft J. Phylogeny and diversification of the largest avian radiation. Proc Natl Acad Sci U S A. 2004;101(30):11040–5. doi: 10.1073/pnas.0401892101 15263073 PMC503738

[71] Oliveros CH, Field DJ, Ksepka DT, Barker FK, Aleixo A, Andersen MJ, et al. Earth history and the passerine superradiation. Proc Natl Acad Sci U S A. 2019;116(16):7916–25. doi: 10.1073/pnas.1813206116 30936315 PMC6475423

[72] Price-Waldman RM, Shultz AJ, Burns KJ. Speciation rates are correlated with changes in plumage color complexity in the largest family of songbirds. Evolution. 2020;74(6):1155–69. doi: 10.1111/evo.13982 32333393

[73] You L, Nishio K, Kowata K, Horikawa M, Fukuchi H, Ogoshi M, et al. Revisiting the hormonal control of sexual dimorphism in chicken feathers. Gen Comp Endocrinol. 2024;357:114601. doi: 10.1016/j.ygcen.2024.114601 39179122

[74] Oribe E, Fukao A, Yoshihara C, Mendori M, Rosal KG, Takahashi S, et al. Conserved distal promoter of the agouti signaling protein (ASIP) gene controls sexual dichromatism in chickens. Gen Comp Endocrinol. 2012;177(2):231–7. doi: 10.1016/j.ygcen.2012.04.016 22554923

[75] Murray JR, Varian-Ramos CW, Welch ZS, Saha MS. Embryological staging of the Zebra Finch, *Taeniopygia guttata*. J Morphol. 2013;274(10):1090–110. doi: 10.1002/jmor.20165 23813920 PMC4239009

[76] Sasai N, Kutejova E, Briscoe J. Integration of signals along orthogonal axes of the vertebrate neural tube controls progenitor competence and increases cell diversity. PLoS Biol. 2014;12(7):e1001907. doi: 10.1371/journal.pbio.1001907 25026549 PMC4098999

[77] Liang Y-C, Wu P, Lin G-W, Chen C-K, Yeh C-Y, Tsai S, et al. Folding Keratin Gene Clusters during Skin Regional Specification. Dev Cell. 2020;53(5):561–576.e9. doi: 10.1016/j.devcel.2020.05.007 32516596 PMC7386462

[78] Bolger AM, Lohse M, Usadel B. Trimmomatic: a flexible trimmer for Illumina sequence data. Bioinformatics. 2014;30(15):2114–20. doi: 10.1093/bioinformatics/btu170 24695404 PMC4103590

[79] O’Leary NA, Wright MW, Brister JR, Ciufo S, Haddad D, McVeigh R, et al. Reference sequence (RefSeq) database at NCBI: current status, taxonomic expansion, and functional annotation. Nucleic Acids Res. 2016;44(D1):D733-45. doi: 10.1093/nar/gkv1189 26553804 PMC4702849

[80] Kim D, Langmead B, Salzberg SL. HISAT: a fast spliced aligner with low memory requirements. Nat Methods. 2015;12(4):357–60. doi: 10.1038/nmeth.3317 25751142 PMC4655817

[81] Pertea M, Kim D, Pertea GM, Leek JT, Salzberg SL. Transcript-level expression analysis of RNA-seq experiments with HISAT, StringTie and Ballgown. Nat Protoc. 2016;11(9):1650–67. doi: 10.1038/nprot.2016.095 27560171 PMC5032908

[82] Pertea M, Pertea GM, Antonescu CM, Chang T-C, Mendell JT, Salzberg SL. StringTie enables improved reconstruction of a transcriptome from RNA-seq reads. Nat Biotechnol. 2015;33(3):290–5. doi: 10.1038/nbt.3122 25690850 PMC4643835

[83] Love MI, Huber W, Anders S. Moderated estimation of fold change and dispersion for RNA-seq data with DESeq2. Genome Biol. 2014;15(12):550. doi: 10.1186/s13059-014-0550-8 25516281 PMC4302049

[84] Kolde R. pheatmap: Pretty Heatmaps. R Package Version 1012. 2019.

[85] Chuong CM, Widelitz RB, Ting-Berreth S, Jiang TX. Early events during avian skin appendage regeneration: dependence on epithelial-mesenchymal interaction and order of molecular reappearance. J Invest Dermatol. 1996;107(4):639–46. doi: 10.1111/1523-1747.ep12584254 8823374

[86] Hu H, Miao Y-R, Jia L-H, Yu Q-Y, Zhang Q, Guo A-Y. AnimalTFDB 3.0: a comprehensive resource for annotation and prediction of animal transcription factors. Nucleic Acids Res. 2019;47(D1):D33–8. doi: 10.1093/nar/gky822 30204897 PMC6323978

[87] Schoch CL, Ciufo S, Domrachev M, Hotton CL, Kannan S, Khovanskaya R, et al. NCBI Taxonomy: a comprehensive update on curation, resources and tools. Database (Oxford). 2020;2020:baaa062. doi: 10.1093/database/baaa062 32761142 PMC7408187

[88] Kumar S, Stecher G, Li M, Knyaz C, Tamura K. MEGA X: Molecular Evolutionary Genetics Analysis across Computing Platforms. Mol Biol Evol. 2018;35(6):1547–9. doi: 10.1093/molbev/msy096 29722887 PMC5967553

[89] Wheeler B, Torchiano M. lmPerm: Permutation Tests for Linear Models. R package version 2.1.0. 2022; https://CRAN.R-project.org/package=lmPerm

