# Supplementary figures and images for: Molecular insights into region-specific sexual dichromatism: Comparative transcriptome analysis of red cheek pigmentation in zebra finches
Source: PLoS Genet. 2025 May 12;21(5):e1011693. doi: 10.1371/journal.pgen.1011693 (PMC12068594; doi:10.1371/journal.pgen.1011693)

S1 Fig

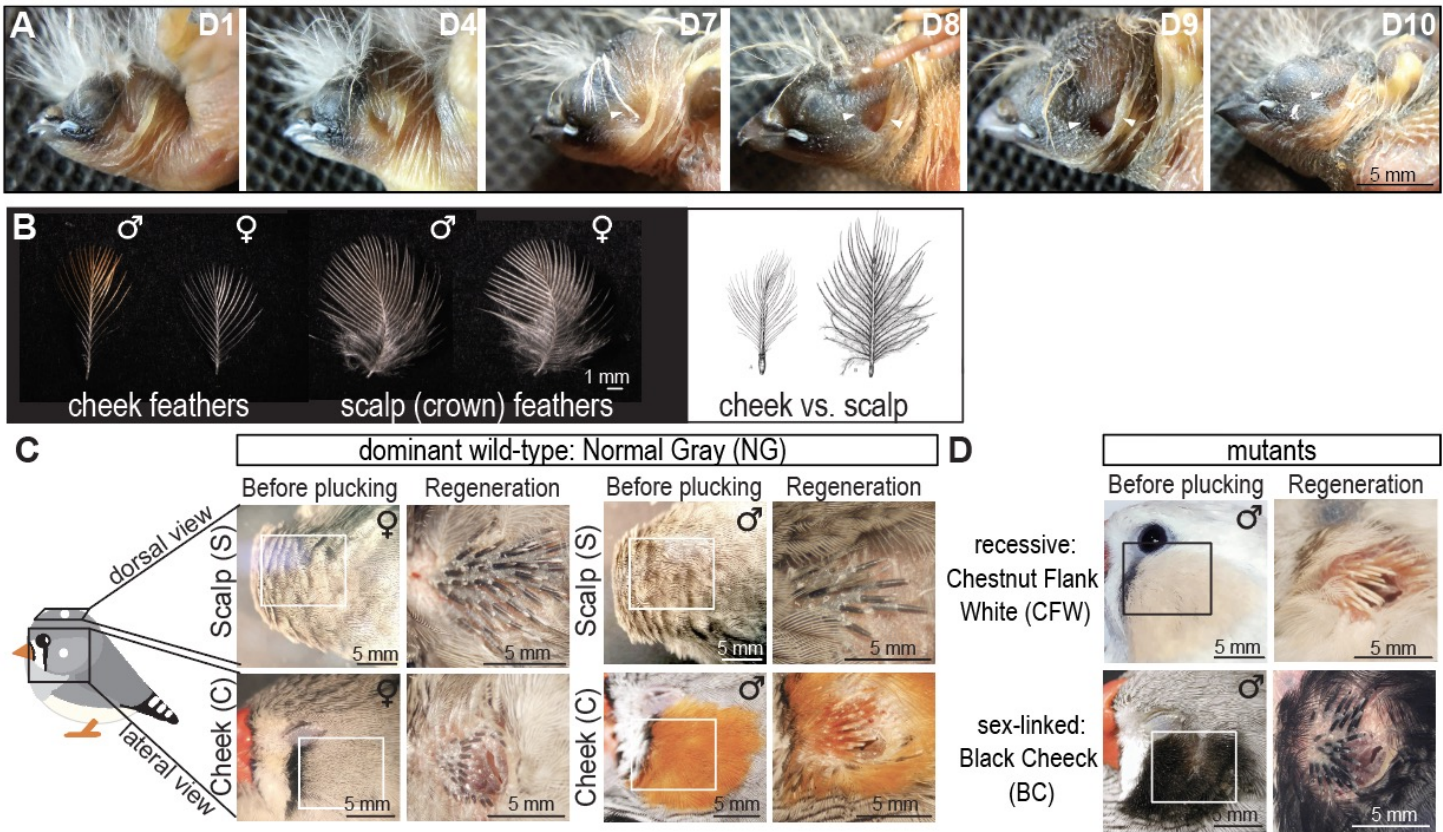

Supplement: S1 Fig — (A) Depiction of the progression of feather growth on juvenile heads from post-hatching day1–10 (D1 to D10). Arrowheads indicate the emerging feather follicles beneath or above the skin. (B) A comparison of the distinct morphology and color between the cheek versus the scalp feathers (the top of head, also referred to as the crown) [34]. Notably, the scalp feather exhibits longer barbs as compared to the cheek feather. (C, D) The strategy for collecting regenerated feathers from the cheek domain (C) and the scalp domain (S) of zebra finches includes three basic types of genetic traits: dominant, recessive, and sex-linked. The dominant wild-type phenotype is called Normal Gray (NG), characterized by males with red cheek and females with gray cheeks. The recessive mutant phenotype, featuring black cheeks, is called Black Cheek (BC), while the sex-linked mutant phenotype, characterized by light red cheeks, is called Chestnut Flank White (CFW). (PDF) [file pgen.1011693.s001.pdf]

S4 Fig

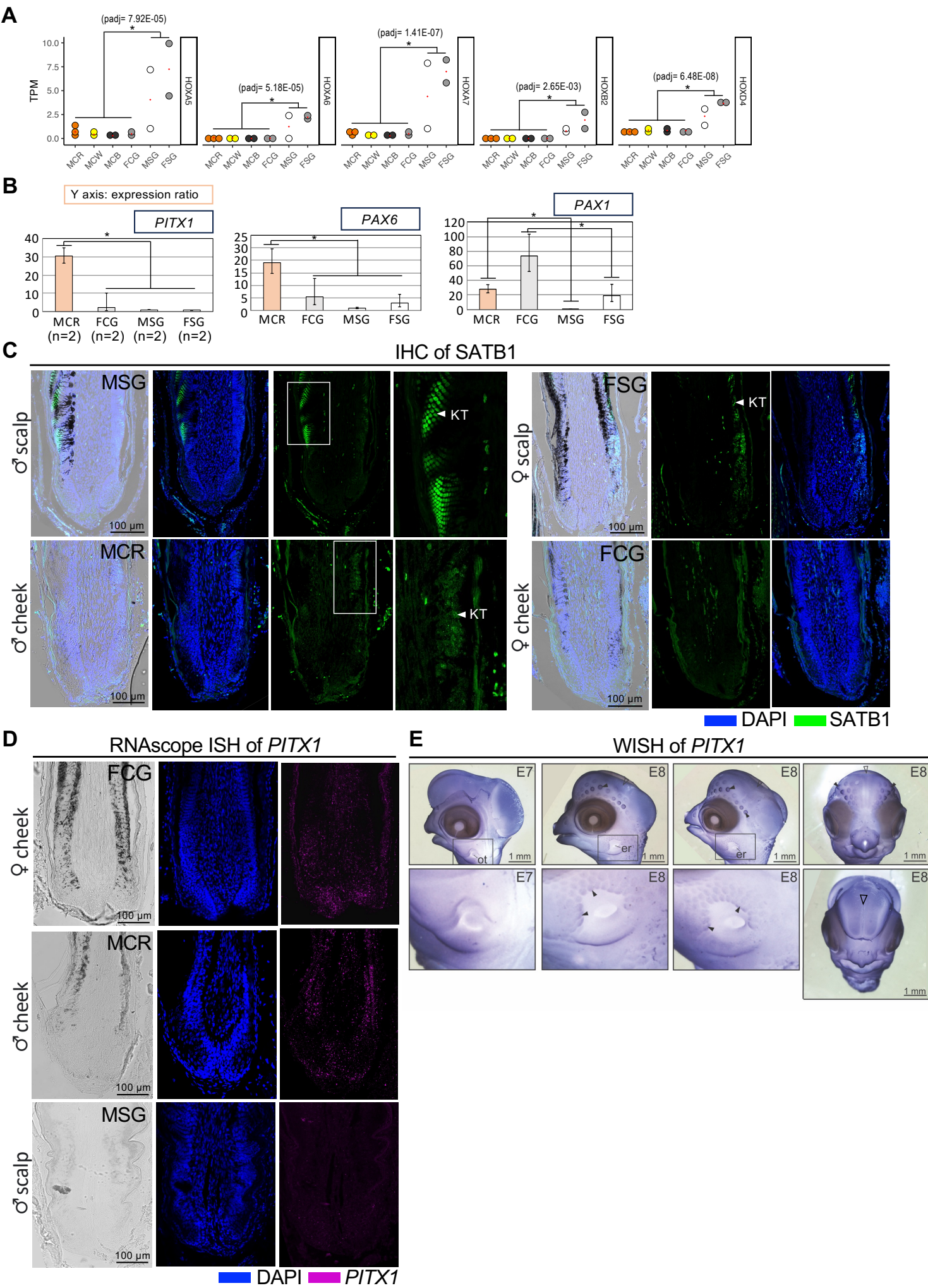

Supplement: S4 Fig — (A) Expression of other representative HOX genes exhibiting high TPM levels in scalps. (B) qPCR validation of genes with high TPM levels in cheeks as shown in Fig 3C. (C) Immunohistochemistry using SATB1 antibody. SATB1 proteins are predominantly expressed in the nuclei of keratinocyte (KT) within scalp feathers as opposed to cheek feathers. (D) RNAscope analysis utilizing PITX1 probes shows that PITX1 mRNA is randomly distributed within cheek feathers, while no PITX1 mRNA is detected in scalp feathers. (E) Whole-mount in situ hybridization employing PITX1 probes on finch embryonic day 7 and 8 (E7 and E8). Preferential expression of PITX1 is observed within developing feather buds on the cheek (see magnified panels below) and eyebrow regions from E8 (arrowheads), compared to scalp region (hollow arrowheads). Abbreviation: er, ear hole; ot, otic vesicle. (PDF) [file pgen.1011693.s004.pdf]

S5 Fig

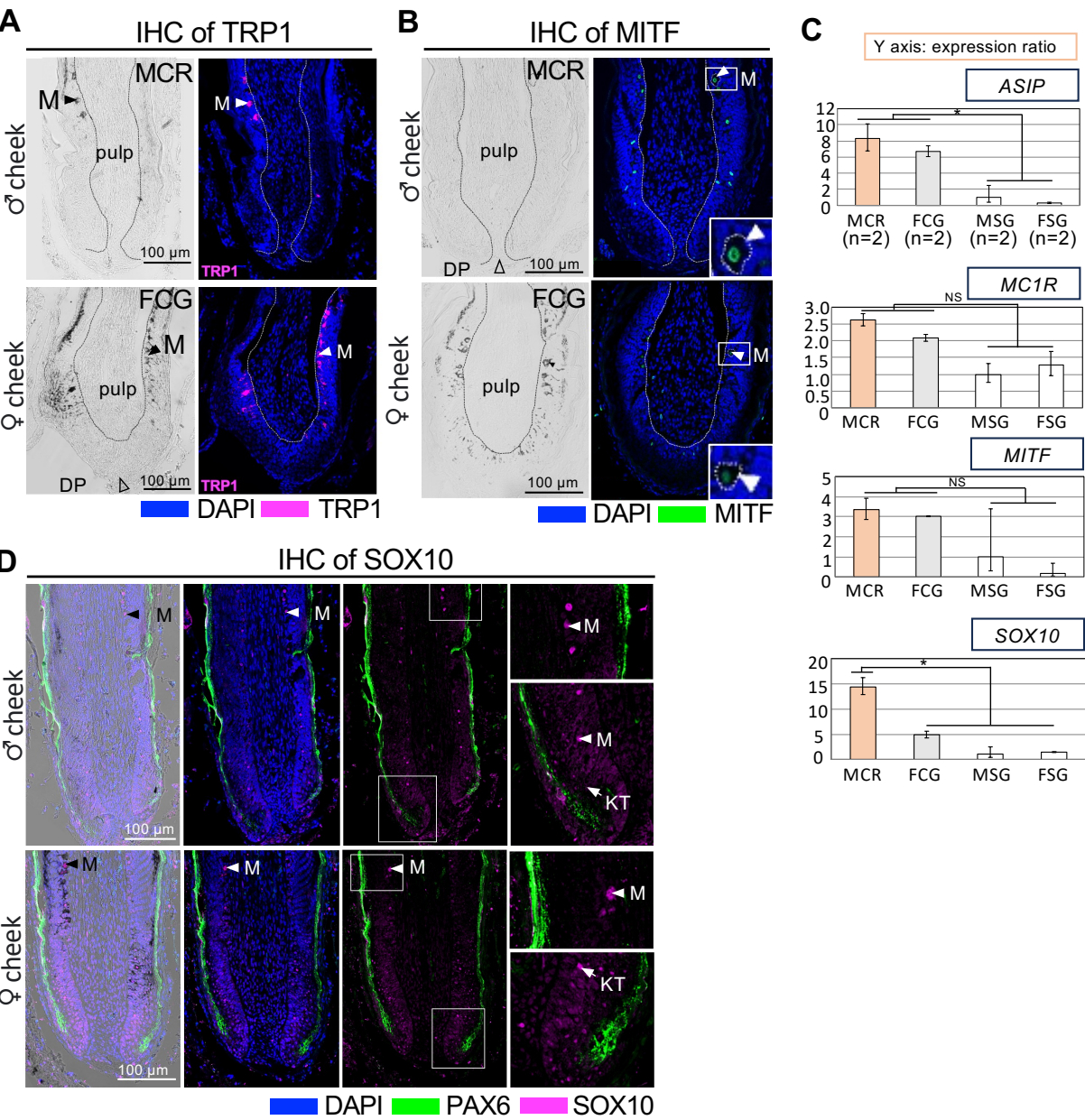

Supplement: S5 Fig — (A) TRP1 proteins are detected in the cytoplasm of certain melanocytes engaged in eumelanin synthesis. (B) Similar numbers and distributions of melanocyte nuclei, which were detected by MITF antibody, within male and female cheek feathers. (C) qPCR validation of ASIP, MC1R, MITF, and SOX10 genes. (D) SOX10 proteins are expressed in melanocytes and partial keratinocytes of both male and female cheeks. Abbreviation: KT, keratinocytes; M, melanocytes. (PDF) [file pgen.1011693.s005.pdf]

S6 Fig

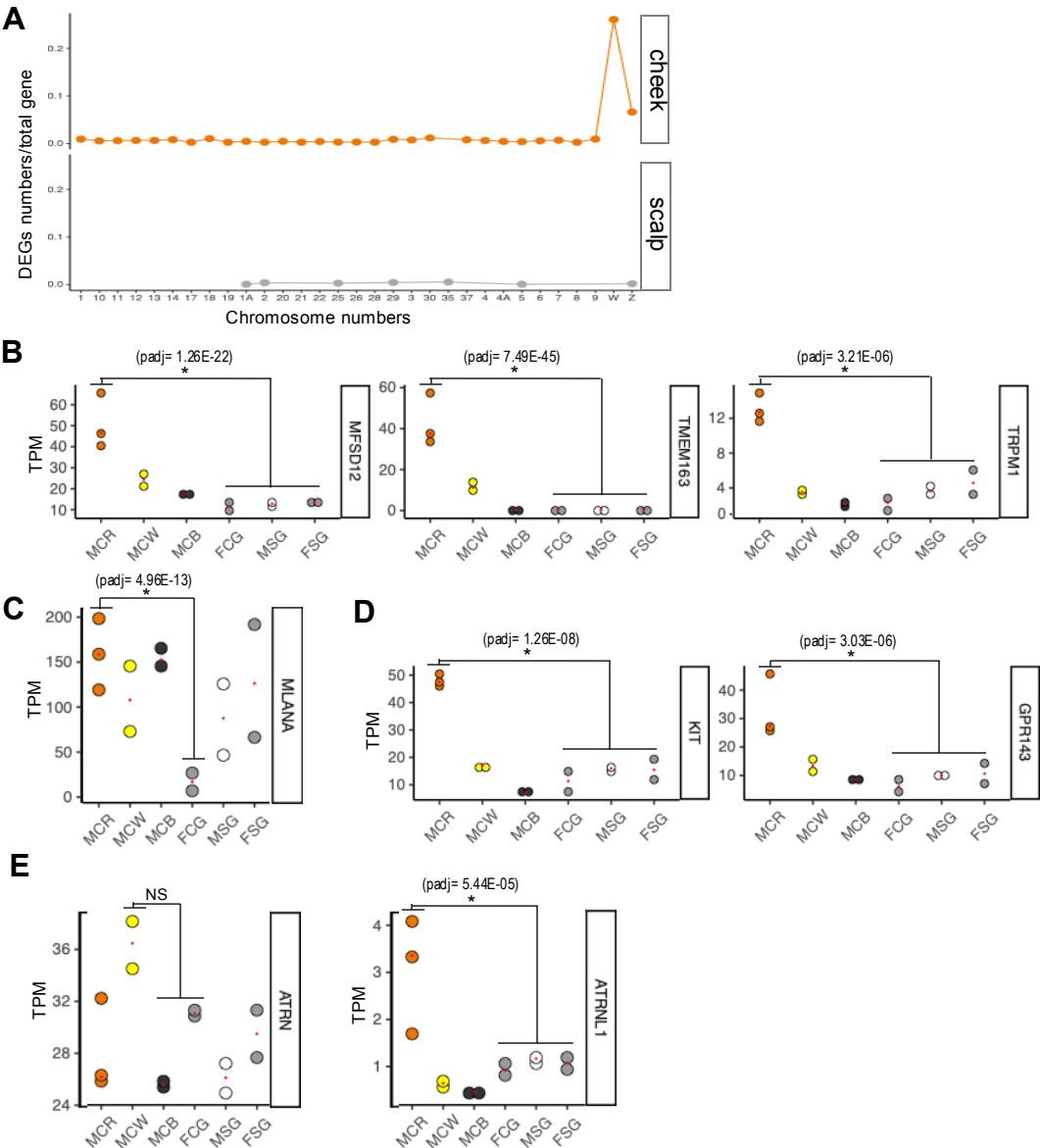

Supplement: S6 Fig — (A) Distribution of DEGs from the cheek comparison (orange dots, MCR vs. FCG) and scalp comparison (gray dots, MSG vs. FSG) across chromosomes. (B–E) TPM levels of genes encoding transporters (B), MLANA (C), receptors (D), and ATRN and ATRNL1 (E). (PDF) [file pgen.1011693.s006.pdf]
