# Supplementary material for: Molecular insights into region-specific sexual dichromatism: Comparative transcriptome analysis of red cheek pigmentation in zebra finches
Source: PLoS Genet. 2025 May 12;21(5):e1011693. doi: 10.1371/journal.pgen.1011693 (PMC12068594; doi:10.1371/journal.pgen.1011693)
Supplement: S2 Fig — (A) Single Comb White Leghorn chicken Gallus gallus. (A’) Auricular tract of Single Comb White Leghorn chicken. (B) Common pigeon. These images are adapted from Figures 53, 55, and 59 in Chapter 3 of the book Avian Anatomy: Integument, 1972. U.S. Agricultural Research Service; U.S. Govt. Print. Off. https://www.google.com.tw/books/edition/Avian_Anatomy_Integument/phJDrhR7RS8C?hl [34]. (PDF) [file pgen.1011693.s002.pdf]

S2 Fig

A

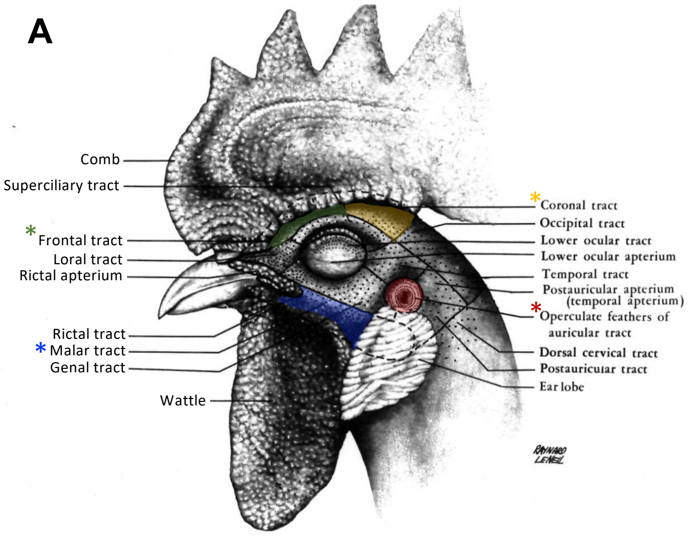

B

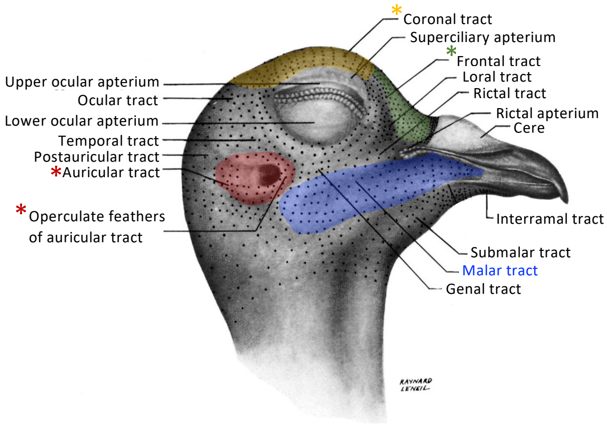

A'

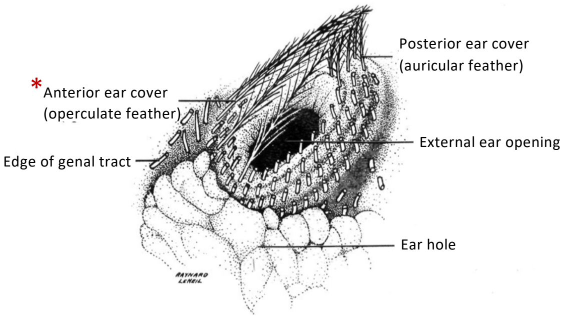

(Images are adapted and modified Figures 53, 55, and 59 from the book Avian Anatomy: Integument, 1972. U.S. Agricultural Research Service; U.S. Govt. Print. Off.) https://books.google.com.tw/books?id=DYIWAAAAYAAJ&printsec=frontcover&hl=zh-TW#v=onepage&q=track&f=false
