# Supplementary material for: Molecular insights into region-specific sexual dichromatism: Comparative transcriptome analysis of red cheek pigmentation in zebra finches
Source: PLoS Genet. 2025 May 12;21(5):e1011693. doi: 10.1371/journal.pgen.1011693 (PMC12068594; doi:10.1371/journal.pgen.1011693)
Supplement: S3 Fig — The comparisons between different domains (scalp and cheek) are presented in (A, B), between cheek feather colors is displayed in (C–F), between sexes is shown in (G), and between male cheek red (MCR) feathers and others is exhibited in (H). Genes exhibiting statistically significant changes (padj < 0.05, log2 Fold Change > 1 or <-1) are marked as red (log2 Fold Change > 1) or blue dots (log2 Fold Change < -1), respectively. The number of differentially expressed genes (DEGs) in each panel is indicated in the upper left or right corner. The significant DEGs for each comparison are listed in S1 File. (PDF) [file pgen.1011693.s003.pdf]

S3 Fig

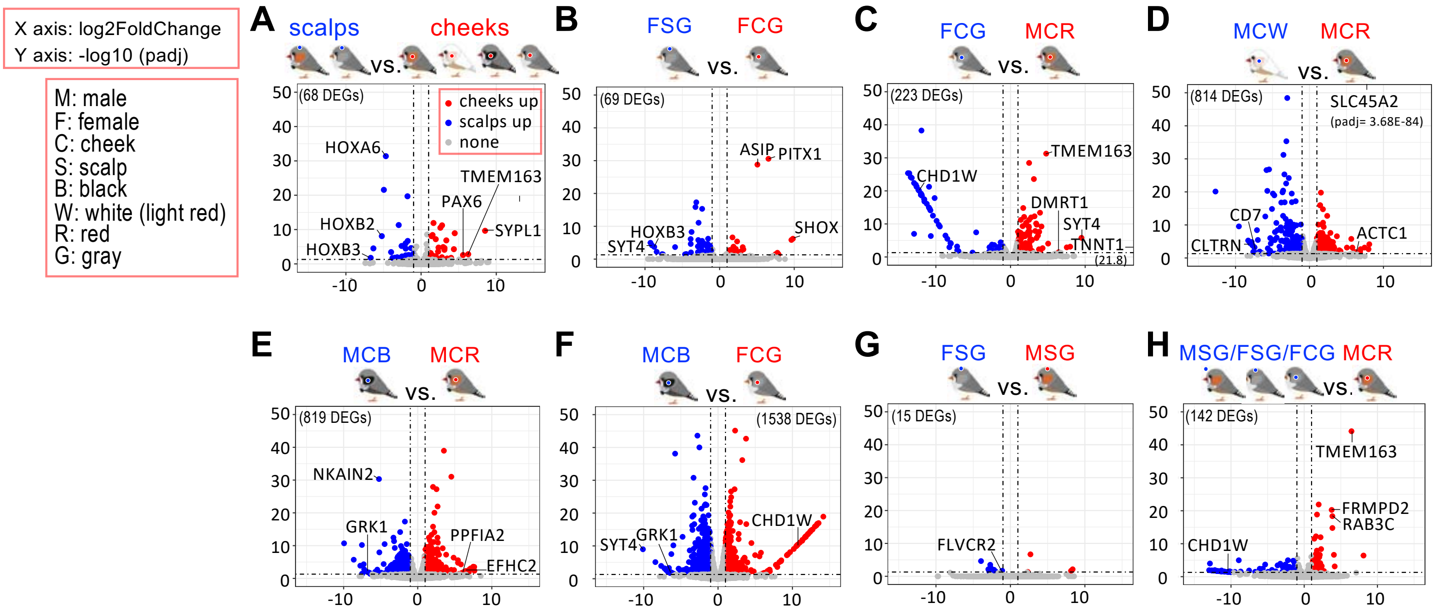

X axis: log2FoldChange
Y axis: -log10 (padj)
M: male
F: female
C: cheek
S: scalp
B: black
W: white (light red)
R: red
G: gray
A scalps cheeks vs.
(68 DEGs)
cheeks up
scalps up
none
HOXA6
HOXB2
HOXB3
TMEM163
PAX6
SYPL1
B FSG FCG vs.
(69 DEGs)
ASIP
PITX1
HOXB3
SYT4
SHOX
C FCG MCR vs.
(223 DEGs)
CHD1W
TMEM163
DMRT1
SYT4
TNNT1
(21.8)
D MCW MCR vs.
(814 DEGs)
SLC45A2
(padj= 3.68E-84)
CD7
CLTRN
ACTC1
E MCB MCR vs.
(819 DEGs)
NKAIN2
GRK1
PPFIA2
EFHC2
F MCB FCG vs.
(1538 DEGs)
CHD1W
SYT4
GRK1
G FSG MSG vs.
(15 DEGs)
FLVCR2
H MSG/FSG/FCG MCR vs.
(142 DEGs)
TMEM163
FRMPD2
RAB3C
CHD1W
